# Divergence in rates of phenotypic plasticity among ectotherms

**DOI:** 10.1111/ele.14147

**Published:** 2022-11-30

**Authors:** Sigurd Einum, Tim Burton

**Affiliations:** ^1^ Department of Biology, Centre for Biodiversity Dynamics Norwegian University of Science and Technology Trondheim Norway; ^2^ Norwegian Institute for Nature Research Trondheim Norway

**Keywords:** acclimation rate, body size, comparative, critical, exponential decay, fitness, phenotypic plasticity, selection, temperature, time course

## Abstract

An individual's fitness cost associated with environmental change likely depends on the rate of adaptive phenotypic plasticity, and yet our understanding of plasticity rates in an ecological and evolutionary context remains limited. We provide the first quantitative synthesis of existing plasticity rate data, focusing on acclimation of temperature tolerance in ectothermic animals, where we demonstrate applicability of a recently proposed analytical approach. The analyses reveal considerable variation in plasticity rates of this trait among species, with half‐times (how long it takes for the initial deviation from the acclimated phenotype to be reduced by 50% when individuals are shifted to a new environment) ranging from 3.7 to 770.2 h. Furthermore, rates differ among higher taxa, being higher for amphibians and reptiles than for crustaceans and fishes, and with insects being intermediate. We argue that a more comprehensive understanding of phenotypic plasticity will be attained through increased focus on the rate parameter.

## Graphical abstract

An individual's fitness cost associated with environmental change likely depends on the rate of adaptive phenotypic plasticity, and yet our understanding of plasticity rates in an ecological and evolutionary context remains limited. We provide the first quantitative synthesis of existing plasticity rate data, focusing on acclimation of temperature tolerance in ectothermic animals, where we demonstrate applicability of a recently proposed analytical approach. We show how rates differ among taxa, being higher for amphibians and reptiles than for crustaceans and fishes, and with insects being intermediate.

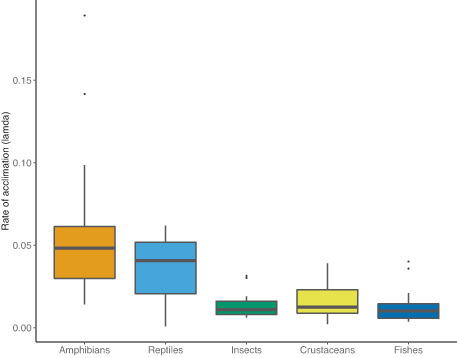

## INTRODUCTION

Phenotypic plasticity, or the expression of different phenotypes across environments by a single genotype, is an important process by which organisms can minimize environmental impacts on fitness (Gabriel, 2005; Gabriel et al., 2005; Padilla & Adolph, 1996; Siljestam & Östman, 2017). Such plasticity can be described by two parameters. First, the *capacity for plasticity* determines the amount by which an individual can actively change its phenotype following a shift in the environment. The term “actively change” is used here to separate between passive and active phenotypic plasticity. An example of passive plasticity is the increase in respiration that typically occurs in response to an increase in temperature, which organisms may counteract over time by active plasticity responses (Kielland et al., 2017). Thus, it is active plasticity that is of interest when studying the responses that organisms have evolved to minimize the effect of environmental change on fitness. This parameter can be measured as the change in the slope of the relationship between trait value and environment (i.e. the slope of the reaction norm) as plasticity proceeds, from acute exposure until the full plastic response has been achieved (see Einum et al., 2019 for arguments why it is this change in the slope, and not slope per se, that describes *capacity for plasticity*).

**FIGURE 1:**
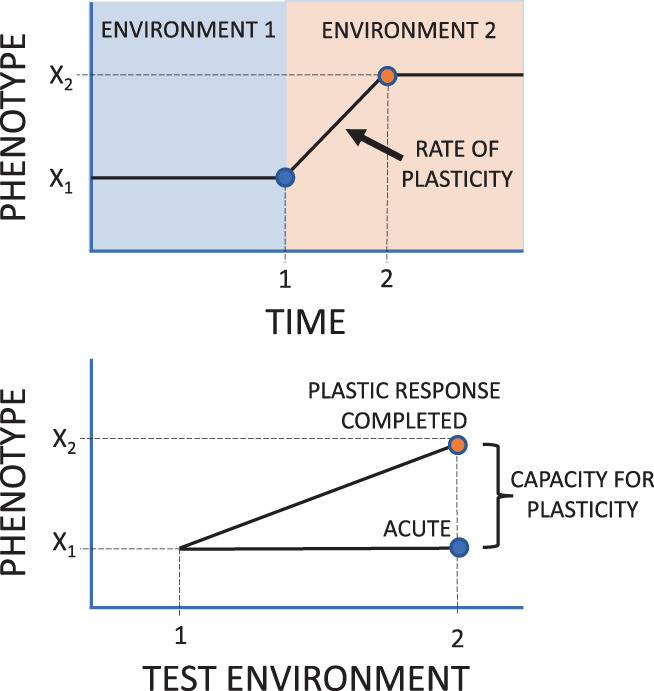
Illustration of the two parameters of phenotypic plasticity. Upper panel shows the change in phenotype over time following a change in the environment at time = 1. The time it takes for the phenotype to become fully adjusted (in this case at time = 2) is determined by the *rate of plasticity*. For this example, the change in phenotype follows a linear trajectory, but other shapes of response are possible (addressed in the current study). Bottom panel shows the corresponding reaction norms for individuals reared in environment 1 acutely exposed to environment 2 (i.e. at time = 1, prior to any adjustment by active plasticity), and for individuals where the active plastic response is completed (i.e. at time = 2). The difference in slope of these two reaction norms represents the *capacity for plasticity* (Einum et al., 2019).

The second parameter of active phenotypic plasticity is the *rate of plasticity*, which represents how quickly the change in phenotype (and hence the change in the reaction norm slope) occurs following a change in the environment (Figure 1). Whereas the *capacity for plasticity* has received considerable theoretical (e.g. Lande, 2014) and empirical interest (e.g. Seebacher et al., 2015; Pottier et al., 2022) from ecologists and evolutionary biologists, empirical support for certain predictions regarding the evolution of this plasticity parameter remain equivocal. For example, while it has been proposed that organisms inhabiting more variable environments should evolve greater *capacity for plasticity*, this is rarely supported by empirical data (Gunderson & Stillman, 2015; Kelly et al., 2012; MacLean et al., 2019; Pereira et al., 2017; Phillips et al., 2016; Sgro et al., 2010; van Heerwaarden et al., 2014, 2016). Recently, Burton et al. (2022) suggested that this discrepancy between theoretical expectations and empirical data gives reason for pause and that greater considerations of the second parameter, the *rate of plasticity*, which addresses the timescale over which plastic phenotypic change occurs, might aid in bringing this field of research forward.

If the plasticity of a trait is an adaptive response, the fitness cost that an organism incurs following a change in its environment should be minimized once the phenotype becomes fully adjusted to the new environment. Hence, the rate at which the phenotype approaches this state should determine how long the individual expresses a sub‐optimal phenotype, and in part, determine the magnitude of the fitness cost associated with that change in the environment. Given that organisms are unlikely to be able to predict changes in all of the relevant environmental variables they are exposed to, it seems plausible that individuals may actually spend a considerable proportion of their time having a phenotype that is not fully adjusted to their current environment. This mismatch between environment and phenotype, and associated cumulative fitness costs, will be exacerbated if the adjustment of the phenotype is slow relative to the timescale of environmental change. Furthermore, as pointed out by Burton et al. (2022), the *rate of plasticity* might even influence how the *capacity for plasticity* evolves because the evolution of capacity depends on the predictability of the environment. Organisms that can rapidly implement their phenotypic response to a new environment can postpone the onset of this process closer to the time of selection in that environment than organisms that do so at a slower rate. Thus, in a temporally autocorrelated environment, a faster *rate of plasticity* might effectively increase predictability in the environment, which in turn should favour the evolution of greater *capacity for plasticity* (Lande, 2014).

Presently, a quantitative synthesis of data on the *rate of plasticity* is lacking, and consideration of how this parameter of phenotypic plasticity might be expected to evolve in response to environmental change is absent from current theoretical models (Lande, 2014; Siljestam & Östman, 2017). Although a substantial number of empirical studies document how phenotypes change over time when introduced into new environments, these studies remain largely descriptive, fail to address evolutionary hypotheses and very rarely (four out of 166 studies surveyed by Burton et al., 2022) attempt to provide any formal statistical quantification of the time course of plasticity. Thus, advancing our understanding of the evolution of phenotypic plasticity might arguably benefit from a shift in focus from *capacity for plasticity* to *rates of plasticity*. To stimulate such a shift, we provide the first comparative analysis of published data describing *rates of plasticity*. In doing so, we follow recent suggestions (Burton et al., 2022) regarding the estimation of plasticity rates in a (i) standardized way, which is (ii) consistent with theory and (iii) directly comparable across taxa and traits.

We draw upon published data from studies of acclimation to temperature among ectotherms. Temperature is an environmental variable that affects all organisms, varies substantially in space and time, and which has particularly pervasive effects on biochemical, physiological and ecological processes in this group of animals (Daufresne et al., 2009). We focus our synthesis on traits describing temperature tolerance. We first determine the shape of how temperature tolerance changes over time (exponential vs. linear decay) in response to a shift in ambient temperature, as this is the first step required when calculating the *rate of plasticity*. After calculating rates of plasticity for each published dataset, we then investigate relationships between *rates of plasticity* and taxonomic class, body size and acclimation temperature. By providing clear evidence that *rates of plasticity* have diverged among ectotherm classes we show how this rate can, and does, evolve, and that increased empirical and theoretical focus on the rate parameter is likely to provide a way forward in understanding evolution of phenotypic plasticity.

## METHODS

### Collection of data

We identified published studies documenting the rate of change in whole‐body measurements of the thermal tolerance of ectotherms in response to manipulation of ambient temperature (Supplementary Information). For most of the papers, data were presented in figure format only, usually as the mean tolerance of groups of individuals that had experienced the ‘new’ temperature for differing lengths of time prior to being measured for the chosen tolerance trait. We digitized such data using the WebPlotDigitizer (https://automeris.io/WebPlotDigitizer/). In addition to thermal tolerance data, we extracted information for the following variables: taxonomic class, species name, mean body mass of individuals used in the experiment, life stage (juvenile vs. adult), acclimation temperature and type of thermal tolerance measure. For the latter variable, a range of different terms that describe behavioural responses to either high or low acute temperature exposure are employed in different studies and are typically taxon‐specific (e.g. knock‐down, loss of equilibrium, paralysis and spasms, loss of righting response). All such endpoints were categorized as a behavioural response. Experiments using some measure of mortality (time until or temperature at which signs of death such as cessation of breath or heart beating occurs) were categorized as a mortality response. For experiments where body mass information was absent we used the following procedure to obtain such data. First, we searched for information on minimum and maximum adult body size (length or mass) for the species in question. If only data on body length were available, we searched for allometries between length and mass for that species, or for species within the same genus, and used these to convert body lengths to mass. We then calculated the mean of the minimum and maximum body mass values. If no data on minimum or maximum size was found, we searched for studies that presented body sizes of adult individuals during field surveys and used the mean of these. The following classes of ectotherms were represented in the dataset: amphibians, reptiles, insects, thecostraca, turbellaria, osteichtyes and malacostraca. For simplicity the latter two will be referred to as fishes and crustaceans, respectively.

### Calculating rates of plasticity

Rates of plasticity were calculated according to the approach outlined by Burton et al. (2022). Thus, for each observation of temperature tolerance at times *t* we first calculated the proportion of the full plastic response that remained to be achieved, *D*_*t*_, as (*z*_*t*_‐ *z*_*∞*_)/(*z*_*0*_ – *z*_*∞*_), where *z*_*0*_ is the first measurement of the phenotype (typically measured prior to the onset of acclimation), *z*_*t*_ is the phenotype after acclimating for a time period *t* (for the current study *t* was standardized to unit h) and *z*_*∞*_ is the fully adjusted phenotype. In theory, *z*_*∞*_ is the final measurement of thermal tolerance. However, given measurement noise this may not be the case. Thus, we defined *z*_*∞*_ as the maximum (for acclimation to higher temperature) or minimum (for acclimation to lower temperature) value for a given measurement of tolerance observed. For each experiment, we considered two potential responses in plasticity of thermal tolerance. First, *D*_*t*_ may have a value of 1 at *t* = 0 (first measurement) and decline linearly with time with a rate *λ*_*L*_ given by *λ*_*L*_ = (1‐*D*_*t*_)/*t* until it reaches 0, after which it will be constant. Alternatively, *D*_*t*_ may have a value of 1 at *t* = 0 and decline as an exponential decay function towards 0 with rate *λ*_*E*_, such that $D_{t}=e^{-\lambda_{E}t}$.

### Statistical analyses – comparison of linear versus exponential decay functions

For each experiment, we fitted both linear and exponential decay models to the observed data. For the linear decline we fitted a piecewise regression with an intercept of 1 (at *t* = 0), which estimates the breakpoint *b* (i.e. the time at which *D*_*t*_ reaches 0), from which the rate *λ*_*L*_ can be calculated as 1/*b*. Thus, both models estimate a single parameter, and their relative fits for a given experiment can be compared directly using their residual standard errors. Models were fitted using the function *nls_multstart* from the *nls.multstart* package v.1.2.0 (Padfield & Matheson, 2020) in R v.4.1.2 (R Core Team, 2021).

For most studies, *z*_*0*_ was measured in individuals prior to transfer to the new temperature. It was often less clear whether experimenters had been able to measure a ‘true’ value of *z*_*∞*_, i.e. thermal tolerance after full acclimation to the new temperature had been obtained. This is a key point when measuring rates of plasticity because estimates of *λ*_*E*_ will be biased if full acclimation to the new environment has not been achieved (Figure S2). However, an advantage of the exponential decay function is that achievement of full acclimation can be assessed by calculating the slope of the estimated function (i.e. $-\lambda_{E}e^{\lambda_{E}t}$) at the final acclimation time point *t*_*n*_ (this slope has an asymptotic value of 0). Thus, this value was calculated for each experiment and included as a covariate in our analysis (see below) to control for any bias introduced by variation in maximum acclimation time among studies.

### Statistical analyses—Variation in rates of phenotypic plasticity

Comparisons of exponential and linear decay models showed that the former was superior to the latter in most cases (see Results). Thus, the following statistical analyses use estimates of *λ*_*E*_ as the dependent variable.

Inspection of the distribution of standard errors (SE) for estimates of *λ*_*E*_ were used to exclude experiments that had poor model fit for the exponential decline models (Figure S3). This was based on judgement, trading‐off maintaining sample size while excluding potentially biased estimates. Using a threshold SE value of 0.01 enabled us to retain 78% of the experiments (*n* = 240 out of 308). The statistical analyses described below use estimates obtained below this threshold. Increasing the threshold SE value to 0.02 increased the inclusion rate to 88% (*n* = 271) without resulting in qualitative changes in the results (Table S1). The bias in estimated *λ*_*E*_ for experiments with SE >0.02 and within the range 0.01–0.02 can be observed by comparing *λ*_*E*_ in experiments within different SE bins (Figure S4).

Variation in *λ*_*E*_ was analysed using meta‐analytical models, fitted using the function *rma.mv* in the package *metafor* v.3.8–1 (Viechtbauer, 2010), while accounting for the sampling variances (i.e. the squared standard error of the *λ*_*E*_ estimates). The full model contained the fixed effects of taxonomic class, body mass, acclimation temperature, type of thermal tolerance measure and slope of the estimated exponential decay function at *t*_*n*_. Life stage was given in less than one third of the identified experiments and was, therefore, not included in the model. Random effects included effects of species, study and experiment. The latter was included because up to several experiments were included per species and study, and meta‐analytical models do not contain an error term. In addition, we included the possibility for a phylogenetic signal in *λ*_*E*_ beyond that imposed by taxonomic class. This was done by building a tree using the package *rotl* v.3.0.12 (Michonneau et al., 2016) and the Open Tree of Life (Open Tree et al.), which was made ultrametric using the function *compute.brlen* in package *ape* v.5.6–2 (Paradis & Schliep, 2019). This tree was used to compute the species relatedness variance–covariance matrix which was included as a second species‐level random effect. Thus, this model accounts for heterogeneity in estimated *λ*_*E*_ due to differences between species unrelated to phylogeny, and a random species effect that accounts for the influence of phylogenetic relatedness within taxonomic classes. All statistical analyses were conducted in R v.4.1.2 (R Core Team, 2021).

The evidence for the full model relative to simplified ones was evaluated by comparing AICc values. We first evaluated the relative support for a model that included the phylogenetic random effect against one that did not contain this term (fitted using REML). After choosing the appropriate random effect structure we proceeded by comparing the relative support for all alternative models (fitted using ML) with simpler fixed structures using the function *dredge* from the package *MuMIn* v.1.47.1 (Barton, 2022). Since we found no evidence for a body mass effect (see Results), and several experiments lacked this information (Table S2), we repeated the comparison of models with different fixed‐effect structures while excluding the body mass variable to allow for inclusion of more experiments in the analysis. Finally, we present the estimated model parameters from the model receiving the strongest support (fitted using REML). For final models, we calculated the heterogeneity *I* ^2^ and pseudo‐*R* ^2^ values. The latter was calculated as the proportional reduction in variance attributed to random effects for models refitted without fixed effects. For all analyses, inspection of residual plots suggested that assumptions of their normality and homogeneity were satisfied.

## RESULTS

### Comparison of linear versus exponential decay functions

A total of 290 experiments could be fitted with both piecewise regression and exponential decay models (Figure S5). The remaining 18 could not be fitted with piecewise regression, which most frequently occurred because the minimum value of *D* (i.e. complete acclimation) was reached at the second observation, in which case multiple breakpoints can yield an identical fit. Of these 290 experiments, comparisons of their residual standard errors revealed that the data in 62% (*n* = 180) of cases was best explained by the exponential decay model. Furthermore, superior fit by piecewise regressions (*N* = 110 experiments) was observed primarily in experiments where thermal tolerance was measured at relatively few time points following exposure of the study organisms to the new temperature (Figure S6). Thus, for more comprehensive experiments (i.e. greater number of measurement points in time), the shape of the plasticity rate response is better described by an exponential decay function. Thus, in all further analyses we use the estimates of *λ*_*E*_ (Table S2).

### Variation in rates of phenotypic plasticity

For one experiment the estimate of *λ*_*E*_ was negative, and six experiments lacked information on acclimation temperature (Table S2). These experiments were excluded from all subsequent analyses. Additionally, the classes thecostraca and turbellaria were represented by only one and two species, respectively (Table S2) and were, therefore, also excluded from subsequent analyses. Finally, for the first set of analyses where we allowed for the inclusion of a body mass effect, an additional 10 experiments that lacked such data (Table S2) were excluded. Thus, the dataset used for these analyses that had an SE of *λ*_*E*_ of less than 0.01 consisted of 219 experiments from a total of 84 species (12 insects, 6 crustaceans, 39 amphibians, 9 reptiles and 18 fishes). Species‐specific mean (SD) estimated *λ*_*E*_ of this dataset was 0.0345 (0.0305) h^−1^, and ranged from 0.0009 to 0.1892 h^−1^. When comparing the fit of models with and without a random phylogenetic effect, the evidence for such an effect was weak (AICc = −899.15 and −901.05 for models with and without, respectively). Thus, we proceeded with comparison of models with different fixed structures while including the random effects of species, study and experiment. Variation in *λ*_*E*_ was best explained by a model that included taxonomic class, acclimation temperature, the slope of the estimated exponential decay function at *t*_*n*_, and the type of thermal tolerance measure, whereas evidence for an effect of body mass was weak (Table 1). We, therefore, repeated the comparison of models with different fixed effects while excluding body mass from the full model. This allowed us to include experiments that lacked body size information, increasing the total number of experiments to 229, representing 91 species (13 insects, 7 crustaceans, 44 amphibians, 9 reptiles and 18 fishes). Species‐specific mean (SD) estimated *λ*_*E*_ of this dataset was 0.0347 (0.0302) h^−1^, with the same range as in the previous dataset. Strong evidence was found for effects of taxonomic class, acclimation temperature and the slope of the estimated exponential decay function at *t*_*n*_, whereas removing the effect of type of thermal tolerance measure from the model caused a slight decrease in AICc (Table 1).

**TABLE 1:** AICc comparisons of the top candidate models explaining variation in rates of plasticity (*λ*_*E*_) in temperature tolerance among different classes of ectothermic animals. ‘Class’ is taxonomic class, ‘Slope’ is the slope of the estimated exponential decay function at the final measurement, ‘Measure’ is the measurement type of thermal tolerance (behaviour vs. mortality), and ‘Mass’ is body mass. Species identity, study and observation are included as random intercepts in all models. ΔAIC_C_ = 12.8 and 14.6 for the best model among those not listed for the top and bottom dataset, respectively

	K	AIC_C_	ΔAIC_C_	w_i_
Reduced dataset containing body mass data				
Acclimation temperature + Class + Slope + Measure	11	−1044.2	0.00	0.378
Acclimation temperature + Class + Slope	10	−1044.0	0.19	0.343
Acclimation temperature + Class + Slope + Measure + Mass	12	−1042.5	1.77	0.156
Acclimation temperature + Class + Slope + Mass	11	−1042.0	2.28	0.121
Full dataset including experiments lacking body mass data				
Acclimation temperature + Class + Slope	10	−1095.0	0.00	0.541
Acclimation temperature + Class + Slope + Measure	11	−1094.7	0.34	0.458

**FIGURE 2:**
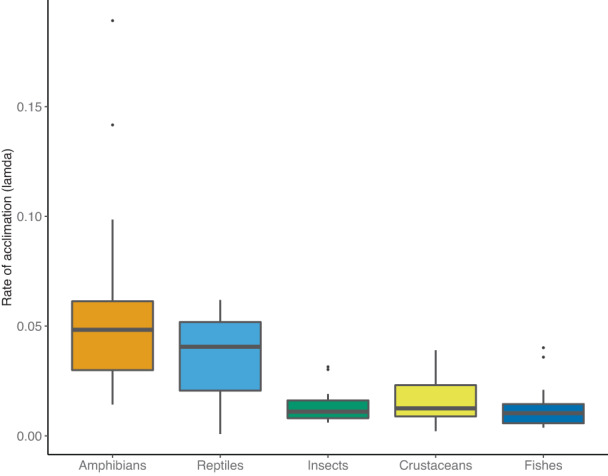
Median observed rates of plasticity (*λ*_*E*_) in temperature tolerance for different classes of ectotherms animals. Data used represent calculated mean observed values for each species. Boxes represent the 25th and 75th percentiles, and whiskers represent 1.5 inter‐quartile range from the box. Estimates obtained from a linear mixed‐effect model that controls for random effects as well as acclimation temperature and the slope of the estimated exponential decay function at the final measurement are given in Table 2.

**FIGURE 3:**
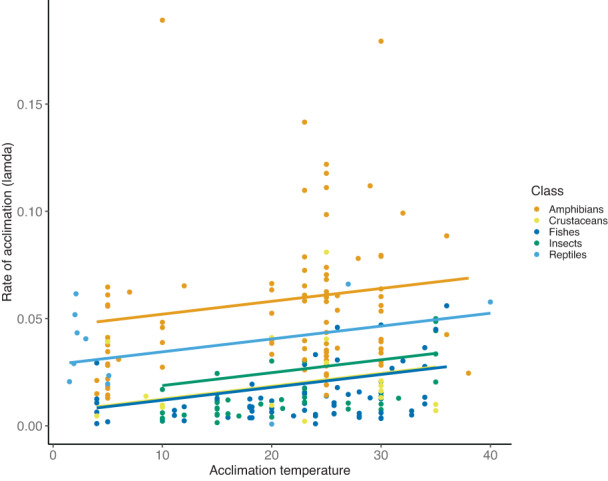
Observed rates of plasticity (*λ*_*E*_) in temperature tolerance as a function of acclimation temperature for different classes of ectothermic animals. Points represent separate experiments, and regression lines with a common slope are based on the best fitting model (Table 2) and are given for the median slope of the estimated exponential decay function at the final acclimation time point.

Inspection of coefficients from the best ranked model showed that plasticity rates were highest among amphibians and reptiles, lowest among fishes and crustaceans and intermediate in insects (Table 2). This pattern persisted when examining distributions of observed *λ*_*E*_ among these classes without correcting for the covariates fitted in the best ranked model (Figure 2). We also observed that plasticity rates increased with acclimation temperature (Table 2, Figure 3). Finally, plasticity rates were observed to be higher when the slope of the estimated exponential decay function at *t*_*n*_ was shallower (Table 2). In other words, experiments where complete acclimation was more likely to have been obtained were associated with higher estimated plasticity rates. This pattern was mainly driven by the amphibian data, which had a large number of experiments with relatively steep slopes at *t*_*n*_ (Figure S7). This is in the opposite direction to what might be predicted if a relationship between this slope and estimated acclimation rate is a statistical artefact (Figure S2). Rather, the causality of this relationship is likely in the opposite direction, i.e. experiments on species that had a low acclimation rate were stopped *before* complete acclimation to the new temperature had occurred. Nevertheless, to evaluate if this anomaly could have influenced our results, we repeated the model comparison summarized in Table 1 but including only experiments where the slope of the estimated exponential decay function at *t*_*n*_ was larger than −0.002 and −0.001, respectively. In both cases, strong evidence was found for effects of taxonomic class, acclimation temperature and the slope of the estimated exponential decay function at *t*_*n*_ (Tables S3, S4). Parameter estimates for the different taxonomic classes showed only minor quantitative changes in comparison with the main analysis (Table S5, S6 vs. Table 2).

**TABLE 2:** Summary of the best fitting model for the full dataset (i.e. including experiments without body mass data, Table 1, re‐fitted using REML) describing variation in rates of plasticity (*λ*_*E*_) in thermal tolerance among different classes of ectothermic animals. ‘Slope at end’ is the slope of the estimated exponential decay function at the final measurement. The amount of variance unaccounted for by fixed effects (*I* ^2^, %) explained by the different random effects are given (total *I* ^2^ = 99.9%). Pseudo‐*R* ^2^ for the model was 0.18

Fixed effects	Estimate	95% CI
Amphibians	0.046	0.033 to 0.059
Reptiles	0.029	0.007 to 0.050
Insects	0.013	−0.003 to 0.028
Fishes	0.006	−0.007 to 0.019
Crustaceans	0.007	−0.012 to 0.025
Acclimation temperature	0.0006	0.0003 to 0.0009
Slope at end	9.12	6.50 to 11.75
Random effects	SD	*I* ^2^
Study	0.0001	18.5
Species	0.0003	46.3
Observation	0.0002	35.1

## DISCUSSION

Here, we provide the first quantitative synthesis of plasticity rates (*λ*) across taxa and apply a novel method to obtain standardized and thus comparable measures of this plasticity parameter. For our focal trait, acclimation of temperature tolerance to temperature change, the shape of the plasticity responses was well described by an exponential decay function (i.e. with the rate *λ*_*E*_). In other words, the absolute rate of change in temperature tolerance when an individual is shifted to a new temperature is proportional to the deviance from the phenotype when completely acclimated to that temperature. We thus validate an assumption that has previously been made in theory describing the evolution of phenotypic plasticity (Lande, 2014). In contrast, superior fit by piecewise linear regression was primarily observed in experiments with poor temporal resolution, thus demonstrating the importance of measuring phenotypes at multiple time points in the new environment to determine the optimal model to use when estimating the shape of the plasticity response.

Variation in estimated *λ*_*E*_ among species was considerable. To put these values into perspective they can be translated into half‐times, or how long it takes for the initial deviation from the fully acclimated phenotype to be reduced by 50% after being shifted to a new environment, which is given as ln(2)/*λ*_*E*_. The mean observed *λ*_*E*_ of 0.0347 h^−1^ corresponds to a half‐time of 20.0 h, whereas the minimum and maximum species‐specific *λ*_*E*_, estimated to 0.0009 and 0.1892 h^−1^, correspond to half‐times of 770.2 and 3.7 h, respectively (based on data where SE *λ*_*E*_ < 0.01). Using model estimates (Table 2), half‐times at 20°C when the final slope of the decay function is zero are 12.0, 16.9, 27.7, 36.5 and 38.5 h for amphibians, reptiles, insects, crustaceans and fishes, respectively. Thus, our analyses demonstrate considerable systematic variation in rates of phenotypic plasticity among these taxa. This begs the question of why such variation has evolved. One might speculate that due to the higher heat capacity of water compared with air, fishes (all species) and crustaceans (five out of seven species in our data), which inhabit aquatic environments experience higher temperature stability than taxa inhabiting terrestrial environments. Furthermore, aquatic habitats are often thermally stratified (lentic habitats such as lakes and oceans) or provide cold‐water plumes (rivers), which allows for behavioural thermoregulation under periods of stressful temperatures (Freitas et al., 2016; Harrison et al., 2016; Kurylyk et al., 2015). This may reduce the strength of selection on rapid plasticity compared with in the terrestrial environment occupied by reptiles and insects (all species in our data). Although the amphibians also inhabit aquatic environments (particularly during juvenile life stages), their utilization of thermal refugia in deep or fast flowing water is likely limited. Unfortunately, habitat use is confounded with phylogeny in this dataset, preventing direct analysis of the effect of habitat on rate of plasticity in thermal tolerance. Thus, although we do demonstrate evolutionary divergence in plasticity rates among these taxa, it remains an open question as to whether this pattern results from evolutionary adaptation to environmental conditions. However, this question could be addressed in future work that targets populations or species that experience known and contrasting patterns of environmental variability.

We observed a positive relationship between acclimation temperature and rate of plasticity in temperature tolerance. This pattern may be explained by the general relationship that exists between developmental rate and body temperature in ectotherms, which is driven by the positive effect of temperature on biochemical reactions and metabolic rate (Brown et al., 2004). It may also explain the observation that within a species, acclimation to high temperature is achieved faster than acclimation to low temperature (Burton et al., 2020). A relationship between metabolic rate and the rate of plasticity was previously hypothesized and addressed by Rohr et al. (2018), but in a less direct manner. Specifically, Rohr et al. (2018) argued that the effect of metabolic rate on rates of thermal plasticity in ectotherms should be evident as a negative relationship between body size and plasticity rate because smaller organisms tend to have a higher mass‐specific metabolic rate than larger ones. They did not however, calculate rates of plasticity from experiments that were explicitly designed to do so. Rather, they used data from experiments that measure the phenotype at only two time points (*z*_*0*_ and *z*_*∞*_ in our terminology), and from this inferred how the bias in acclimation capacity caused by insufficient acclimation time was influenced by body size. Based on their results it was concluded that rates of plasticity appeared to be higher for smaller organisms. Using a more direct approach we failed to find support for a general relationship between body size and rate of thermal plasticity. Yet, our observation that the rate of plasticity in temperature tolerance is positively related to acclimation temperature suggests a role for metabolic rate in causing some of the variation in plasticity rate across experiments.

Given the general patterns in rate of plasticity observed here, further efforts in studying this plasticity parameter may be fruitful and provide a better foundation for understanding how plasticity evolves in response to environmental variation. From an empirical perspective, including a temporal‐dimension in experiments that study plasticity may be included without large costs. In this respect, we make two recommendations. First, a proper choice of model (linear vs. exponential decay) for estimating lambda requires multiple measurements of the phenotype as it responds to the new environment. Our analyses indicate that five or more measurements may be required to adequately establish the shape of the plasticity response (Figure S6). Superficially, this requirement might appear to substantially increase the workload of such studies in comparison with studies that only estimate the capacity for plastic phenotypic change in a trait. However, once the model that best describes the shape of the plastic response to the new environment is established, a single measurement *z*_*t*_ after time *t* (which must be prior to achievement of full acclimation) in addition to those typically measured (*z*_*0*_ in non‐acclimated individuals and *z*_*∞*_ after the full acclimation response has been obtained) is sufficient to accurately estimate *D*_*t*_, which in turn can be used to calculate the rate of plasticity (*λ*_*E*_ = ln(*D*_*t*_)/*t* for exponential decay or *λ*_*L*_ = (1‐*D*_*t*_)/*t* for linear decay). Thus, the workload in such experiments can be greatly reduced by performing a pilot experiment with sufficient temporal resolution (in terms of measurement time points) that provides a precise description of the shape of plasticity response to the new environment before performing more replicates at a lower temporal resolution to obtain the desired estimates of *λ*. It should be noted that *λ*_*E*_ and *λ*_*L*_ are not directly comparable because the initial approach towards the fully adjusted phenotype is more rapid under exponential decay. Thus, the relative support for these two types of plasticity responses should be reported. As a second recommendation, experimenters should strive to ensure that complete acclimation to the new environment is achieved prior to measuring *z*_*∞*_. Our analyses show that failing to do so can, and does, lead to bias in estimation of *λ*_*E*_ (Figure S2, S7). Ideally this is achieved by rearing individuals in all the alternative environments for the whole duration of the experiment (i.e. both prior to and after some of the individuals are transferred into new environments). As pointed out by Burton et al. (2022), this has rarely been done in studies of rates of plasticity. Rather, the majority of studies first acclimate the animals to a single initial environment before shifting them to a new environment and then performing repeated measures of phenotype in this new environment for what typically appears to be a pre‐determined (and potentially insufficient) duration.

Natural next steps in research on evolution of plasticity would be to test for links between environmental variation and the evolution of rates of plasticity and to provide theoretical models that address the co‐evolution of plasticity rates and capacity (see Introduction). Although this is beyond the scope of the current paper, our work provides both methodology and novel insights that should stimulate future work along these lines. We also re‐emphasize a point made previously (Burton et al., 2022) – that selection on the rate of plasticity might be stronger than selection on the capacity for plasticity. Evolutionary theory posits a central role for phenotypic plasticity in mitigating the fitness impact of environmental variation but that possessing the potential for such a response is associated with a fitness cost in stable environments (Lande, 2009). Fitness costs of plasticity can be categorized into costs of maintenance and costs of production. Costs of maintenance represent the investment of resources into maintaining the machinery required for detecting and responding to a change in the environment and will be paid at a constant rate independent of environmental conditions (Auld et al., 2010). In contrast, production costs are only paid when the plastic response is triggered and are compensated by the fitness benefits associated with changing the phenotype. If one assumes that the capacity for plasticity can be increased by operating the ‘machinery’ required to change a trait for a longer duration, this will increase production costs but not maintenance costs. Populations living in less variable environments may, therefore, pay a small price for maintaining their capacity for plasticity (as shown by Van Buskirk & Steiner, 2009), and adaptation of this parameter of plasticity to levels of environmental fluctuations may, therefore, be relatively modest in magnitude. In contrast, increasing the rate of change in the same trait would require increasing the size or output of that ‘machinery’, with corresponding increases in maintenance costs. Populations living in less variable environments should, therefore, experience strong selection against maintaining rapid plasticity due to higher maintenance costs, and adaptative evolution across populations may then be expected to be more pronounced for the rate of plasticity. This line of reasoning is also consistent with theoretical results showing that maintenance costs shape the evolution of plasticity to a greater extent than production costs (Sultan & Spencer, 2002). Given these considerations, and the results presented in the current study, it seems prudent to address the hypothesis that adaptation to environmental variation may be more pronounced in terms of rates of plasticity rather than capacity of plasticity. By providing clear evidence that rates of plasticity have diverged among ectotherm classes we show that it is a trait that evolves and that increased empirical and theoretical focus on the rate parameter is likely to provide a way forward for a more comprehensive understanding of phenotypic plasticity.

## AUTHOR CONTRIBUTIONS

Both authors conceived the study and identified data sources, SE extracted the data, conducted the analyses and wrote the first draft of the manuscript, both authors contributed to revisions.

### PEER REVIEW

The peer review history for this article is available at https://publons.com/publon/10.1111/ele.14147.

## Supporting information


Appendix S1:

## Data Availability

Data and code are available at Dryad, https://doi.org/10.5061/dryad.gtht76hqq.

## References

[ele14147-bib-0001] Auld, J.R. , Agrawal, A.A. & Relyea, R.A. (2010) Re‐evaluating the costs and limits of adaptive phenotypic plasticity. Proceedings of the Royal Society B: Biological Sciences, 277, 503–511. 10.1098/rspb.2009.1355 PMC2842679 19846457

[ele14147-bib-0002] Barton, K. (2022) MuMIn: Multi‐model inference. R package version 1.47.1. https://CRAN.R‐project.org/package=MuMIn

[ele14147-bib-0003] Brown, J.H. , Gillooly, J.F. , Allen, A.P. , Savage, V.M. & West, G.B. (2004) Toward a metabolic theory of ecology. Ecology, 85, 1771–1789. Available from: 10.1890/03-9000

[ele14147-bib-0004] Burton, T. , Lakka, H.‐K. & Einum, S. (2020) Acclimation capacity and rate change through life in the zooplankton *daphnia* . Proceedings of the Royal Society B: Biological Sciences, 287, 20200189. Available from: 10.1098/rspb.2020.0189 PMC7209067 32228409

[ele14147-bib-0005] Burton, T. , Ratikainen, I.I. & Einum, S. (2022) Environmental change and the rate of phenotypic plasticity. Global Change Biology, 28, 5337–5345. Available from: 10.1111/gcb.16291 35729070 PMC9541213

[ele14147-bib-0006] Daufresne, M. , Lengfellner, K. & Sommer, U. (2009) Global warming benefits the small in aquatic ecosystems. Proceedings of the National Academy of Sciences, 106, 12788–12793. Available from: 10.1073/pnas.0902080106 PMC2722360 19620720

[ele14147-bib-0007] Einum, S. , Ratikainen, I. , Wright, J. , Pélabon, C. , Bech, C. , Jutfelt, F. et al. (2019) How to quantify thermal acclimation capacity? Global Change Biology, 25, 1893–1894. Available from: 10.1111/gcb.14598 30779405

[ele14147-bib-0008] Freitas, C. , Olsen, E.M. , Knutsen, H. , Albretsen, J. & Moland, E. (2016) Temperature‐associated habitat selection in a cold‐water marine fish. Journal of Animal Ecology, 85, 628–637. Available from: 10.1111/1365-2656.12458 26476092

[ele14147-bib-0009] Gabriel, W. (2005) How stress selects for reversible phenotypic plasticity. Journal of Evolutionary Biology, 18, 873–883. Available from: 10.1111/j.1420-9101.2005.00959.x 16033559

[ele14147-bib-0010] Gabriel, W. , Luttbeg, B. , Sih, A. & Tollrian, R. (2005) Environmental tolerance, heterogeneity, and the evolution of reversible plastic responses. American Naturalist, 166, 339–353. 10.1086/432558 16224689

[ele14147-bib-0011] Gunderson, A.R. & Stillman, J.H. (2015) Plasticity in thermal tolerance has limited potential to buffer ectotherms from global warming. Proceedings of the Royal Society B: Biological Sciences, 282, 20150401. Available from: 10.1098/rspb.2015.0401 PMC4455808 25994676

[ele14147-bib-0012] Harrison, P.M. , Gutowsky, L.F. , Martins, E.G. , Patterson, D.A. , Cooke, S.J. & Power, M. (2016) Temporal plasticity in thermal habitat selection of burbot *Lota lota* a diel‐migrating winter‐specialist. Journal of Fish Biology, 88, 2111–2129. Available from: 10.1111/jfb.12990 27125426

[ele14147-bib-0013] Kelly, M.W. , Sanford, E. & Grosberg, R.K. (2012) Limited potential for adaptation to climate change in a broadly distributed marine crustacean. Proceedings of the Royal Society B: Biological Sciences, 279, 349–356. Available from: 10.1098/rspb.2011.0542 PMC3223665 21653591

[ele14147-bib-0014] Kielland, Ø.N. , Bech, C. & Einum, S. (2017) No evidence for thermal transgenerational plasticity in metabolism when minimizing the potential for confounding effects. Proceedings of the Royal Society B: Biological Sciences, 284, 20162494. Available from: 10.1098/rspb.2016.2494 PMC5247506 28077777

[ele14147-bib-0015] Kurylyk, B.L. , MacQuarrie, K.T.B. , Linnansaari, T. , Cunjak, R.A. & Curry, R.A. (2015) Preserving, augmenting, and creating cold‐water thermal refugia in rivers: concepts derived from research on the Miramichi River, New Brunswick (Canada). Ecohydrology, 8, 1095–1108. Available from: 10.1002/eco.1566

[ele14147-bib-0016] Lande, R. (2009) Adaptation to an extraordinary environment by evolution of phenotypic plasticity and genetic assimilation. Journal of Evolutionary Biology, 22, 1435–1446. 19467134 10.1111/j.1420-9101.2009.01754.x

[ele14147-bib-0017] Lande, R. (2014) Evolution of phenotypic plasticity and environmental tolerance of a labile quantitative character in a fluctuating environment. Journal of Evolutionary Biology, 27, 866–875. 24724972 10.1111/jeb.12360

[ele14147-bib-0018] MacLean, H.J. , Sørensen, J.G. , Kristensen, T.N. , Loeschcke, V. , Beedholm, K. , Kellermann, V. et al. (2019) Evolution and plasticity of thermal performance: an analysis of variation in thermal tolerance and fitness in 22 *drosophila* species. Philosophical Transactions of the Royal Society B: Biological Sciences, 374, 20180548. Available from: 10.1098/rstb.2018.0548 PMC6606468 31203763

[ele14147-bib-0019] Michonneau, F. , Brown, J.W. & Winter, D.J. (2016) Rotl: an R package to interact with the open tree of life data. Methods in Ecology and Evolution, 7, 1476–1481. doi:10.1111/2041-210X.12593 OpenTree et al. Open Tree of Life Synthetic Tree. 10.1111/2041-210X.12593

[ele14147-bib-0020] Padfield, D. & Matheson, G. (2020) Nls.Multstart: robust non‐linear regression using AIC scores. R package version 1.2.0. https://CRAN.R‐project.org/package=nls.multstart

[ele14147-bib-0021] Padilla, D.K. & Adolph, S.C. (1996) Plastic inducible morphologies are not always adaptive: the importance of time delays in a stochastic environment. Evolutionary Ecology, 10, 105–117. Available from: 10.1007/BF01239351

[ele14147-bib-0022] Paradis, E. & Schliep, K. (2019) Ape 5.0: an environment for modern phylogenetics and evolutionary analyses in R. Bioinformatics, 35, 526–528. 30016406 10.1093/bioinformatics/bty633

[ele14147-bib-0023] Pereira, R.J. , Sasaki, M.C. & Burton, R.S. (2017) Adaptation to a latitudinal thermal gradient within a widespread copepod species: the contributions of genetic divergence and phenotypic plasticity. Proceedings of the Royal Society B: Biological Sciences, 284, 20170236. Available from: 10.1098/rspb.2017.0236 PMC5413927 28446698

[ele14147-bib-0024] Phillips, B.L. , Muñoz, M.M. , Hatcher, A. , Macdonald, S.L. , Llewelyn, J. , Lucy, V. et al. (2016) Heat hardening in a tropical lizard: geographic variation explained by the predictability and variance in environmental temperatures. Functional Ecology, 30, 1161–1168. Available from: 10.1111/1365-2435.12609

[ele14147-bib-0025] Pottier, P. , Burke, S. , Zhang, R.Y. , Noble, D.W.A. , Schwanz, L.E. , Drobniak, S.M. et al. (2022) Developmental plasticity in thermal tolerance: ontogenetic variation, persistence, and future directions. Ecology Letters, 25, 2245–2268. Available from: 10.1111/ele.14083 36006770 PMC9804923

[ele14147-bib-0026] R Core Team . (2021) R: a language and environment for statistical computing. Vienna, Austria: R Foundation for Statistical Computing. Available from: https://www.R‐project.org/

[ele14147-bib-0027] Rohr, J.R. , Civitello, D.J. , Cohen, J.M. , Roznik, E.A. , Sinervo, B. & Dell, A.I. (2018) The complex drivers of thermal acclimation and breadth in ectotherms. Ecology Letters, 21, 1425–1439. Available from: 10.1111/ele.13107 30009486

[ele14147-bib-0028] Seebacher, F. , White, C. & Franklin, C. (2015) Physiological plasticity increases resilience of ectothermic animals to climate change. Nature Climate Change, 5, 61–66. Available from: 10.1038/nclimate2457

[ele14147-bib-0029] Sgro, C.M. , Overgaard, J. , Kristensen, T.N. , Mitchelll, K.A. , Cockerell, F.E. & Hoffmann, A.A. (2010) A comprehensive assessment of geographic variation in heat tolerance and hardening capacity in populations of *Drosophila melanogaster* from eastern Australia. Journal of Evolutionary Biology, 23, 2484–2493. Available from: 10.1111/j.1420-9101.2010.02110.x 20874849

[ele14147-bib-0030] Siljestam, M. & Östman, Ö. (2017) The combined effects of temporal autocorrelation and the costs of plasticity on the evolution of plasticity. Journal of Evolutionary Biology, 30, 1361–1371. Available from: 10.1111/jeb.13114 28485061

[ele14147-bib-0031] Sultan, S.E. & Spencer, H.G. (2002) Metapopulation structure favors plasticity over local adaptation. The American Naturalist, 160, 271–283. Available from: 10.1086/341015 18707492

[ele14147-bib-0032] Van Buskirk, J. & Steiner, U.K. (2009) The fitness costs of developmental canalization and plasticity. Journal of Evolutionary Biology, 22, 852–860. Available from: 10.1111/j.1420-9101.2009.01685.x 19226418

[ele14147-bib-0033] van Heerwaarden, B. , Kellermann, V. & Sgrò, C.M. (2016) Limited scope for plasticity to increase upper thermal limits. Functional Ecology, 30, 1947–1956. Available from: 10.1111/1365-2435.12687

[ele14147-bib-0034] van Heerwaarden, B. , Lee, R.F.H. , Overgaard, J. & Sgrò, C.M. (2014) No patterns in thermal plasticity along a latitudinal gradient in *Drosophila simulans* from eastern Australia. Journal of Evolutionary Biology, 27, 2541–2553. Available from: 10.1111/jeb.1251022 25262984

[ele14147-bib-0035] Viechtbauer, W. (2010) Conducting meta‐analyses in R with the metafor package. Journal of Statistical Software, 36, 1–48. Available from: 10.18637/jss.v036.i03

