# Supplementary material for: Divergence in rates of phenotypic plasticity among ectotherms
Source: Ecol Lett. 2022 Nov 30;26(1):147–56. doi: 10.1111/ele.14147 (PMC10099672; doi:10.1111/ele.14147)
Supplement: Supplementary file 1 — Appendix S1: [file ELE-26-147-s001.docx]

**Supplementary Information**

*Divergence in rates of phenotypic plasticity among ectotherms*

Sigurd Einum^1*^ and Tim Burton^1,2^

^1^Centre for Biodiversity Dynamics, Department of Biology, Norwegian University of Science and Technology, Realfagbygget, NO-7491 Trondheim, Norway

^2^Norwegian Institute for Nature Research, PO Box 5685, Torgarden, NO-7485, Trondheim, Norway. *corresponding author: [sigurd.einum@ntnu.no](mailto:sigurd.einum@ntnu.no)

*Procedure for search and selection of data*

Potentially relevant papers were identified using the following procedure. First, we included eight papers identified by Burton et al. (2022) that were based on a search in Web of Science (see Burton et al. for procedure). Second, we conducted two searches on Google Scholar during January 2022. In the first search, we used the terms "temperature" and "rate of acclimation". The 739 hits were sorted by Google Scholar based on relevance, and the first 200 abstracts were scanned for inclusion. In the second search, we used the terms "temperature", "acclimation", "time course" and "critical". This resulted in 30200 hits, which also were sorted based on relevance, and where the first 200 abstracts were scanned for inclusion. From both these searches, decisions of inclusion at this stage were made based on whether the abstract contained information suggesting that the study organisms had been acclimated to an initial temperature for a given period of time, and then transferred to a ‘new’ temperature, after which thermal tolerance was measured on subsamples of these individuals at different points in time. The first search resulted in a total of 55 papers (none of these appeared among the last 30 scanned abstracts), whereas the second search identified an additional seven papers (the majority of relevant hits had been identified in the initial search). Finally, the reference lists of papers identified above were manually scanned for reference to additional papers not located in any of the previous searches where the titles indicated that they might contain the information required as indicated above. This resulted in a further 18 papers. Thus, a total of 88 papers were identified during this procedure. For each of these we obtained the full paper and checked whether it presented the required data in an accessible form. Our selection criterium was that the study presented data on thermal tolerance (measured as critical temperatures (i.e. CTmax, CTmin), time to death/immobility of individuals, or mortality rate at a stressful temperature in groups of organisms) at a minimum of three time points at and following movement from one rearing temperature to another one. Fifty-nine of the 88 papers were found to satisfy these criteria. Many of these contained data from several experiments (different species and/or different acclimation temperatures). A diagram summarizing the search and selection procedure is given in Fig. S1. It should be noted that we did not attempt to follow the PRISMA guidelines for meta-analyses (O’Dea et al., 2021). Our intention was to conduct a more exploratory and descriptive first quantitative synthesis of studies on rates of plasticity, that with a reasonable effort compiled sufficient amounts of data to address our main questions (1) whether the change in temperature tolerance in response to a shift in ambient temperature is best approximated by exponential or linear decay models, and (2) whether there are any phylogenetic patterns in this rate. Since these are novel questions, none of the empirical studies identified in our literature search actually address these questions themselves. Rather, individual studies typically focus on a single or a few highly related species, and data are usually presented in a descriptive manner without any formal statistical quantification of plasticity rates (e.g. less than 3% of studies reviewed by Burton et al., 2022 did this). Thus, there should be no reason for the actual results of a study to influence the decision to publish them. The only exception would be for studies that test for but fail to observe any acclimation response. However, even if such studies may be underrepresented in the literature, rates of acclimation cannot be calculated in such cases and thus these would have been excluded from the current study regardless. We therefore consider the risk of publication bias or procedures used for data collection in influencing our conclusions to be limited.


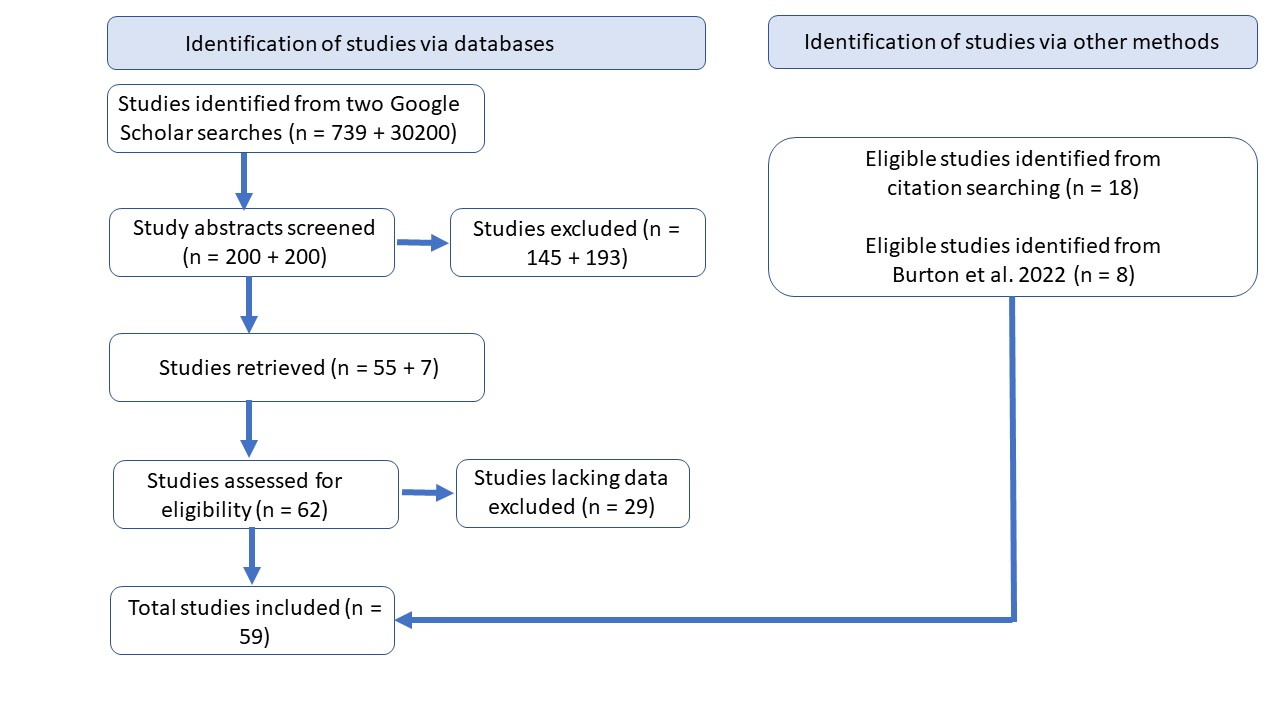


**Fig. S1.** Outline of search and selection process of studies reporting data on rates of acclimation of thermal tolerance.


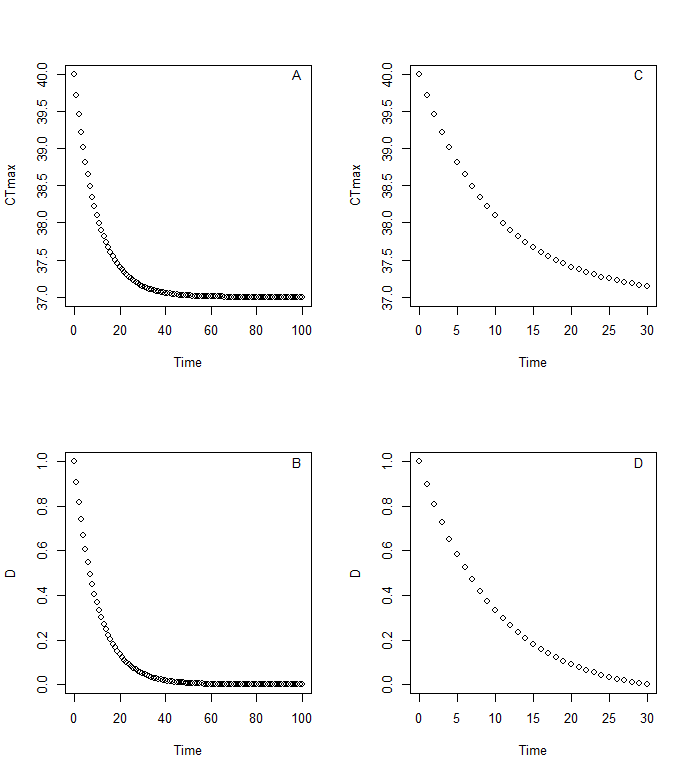


**Fig. S2.** Figures illustrating how incomplete acclimation introduces bias when estimating the rate of acclimation. (A) hypothetical measured critical maximum temperatures for an organism which has *λ_E_* = 0.1. (B) the corresponding calculated values of *D*, which when fitted with an exponential decay function provides an estimated *λ_E_* = 0.100. (C) The same dataset as in A, but only including data up to Time = 30, and (D) the corresponding values of *D* which when fitted with an exponential decay function provides an estimated *λ_E_* = 0.113.


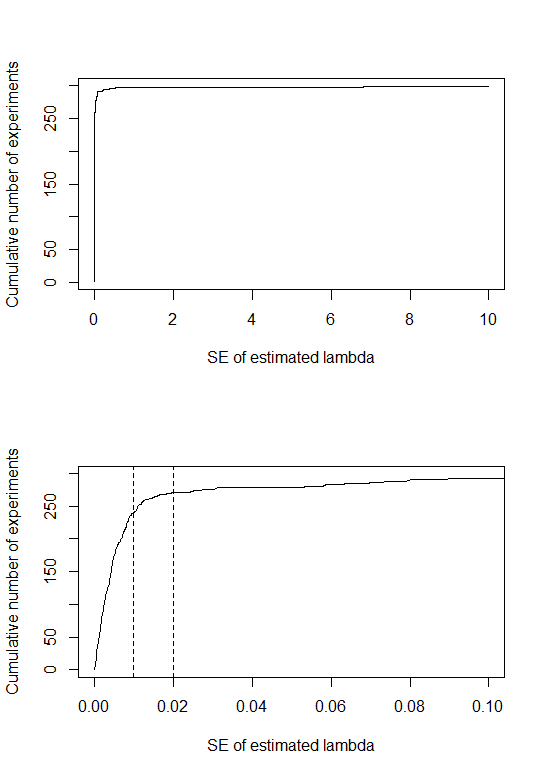


**Fig. S3.** Cumulative distribution of standard errors (SE) in estimates of acclimation rate (*λ_E_*). SE values of 0.01 and 0.02 are indicated by vertical dashed lines.


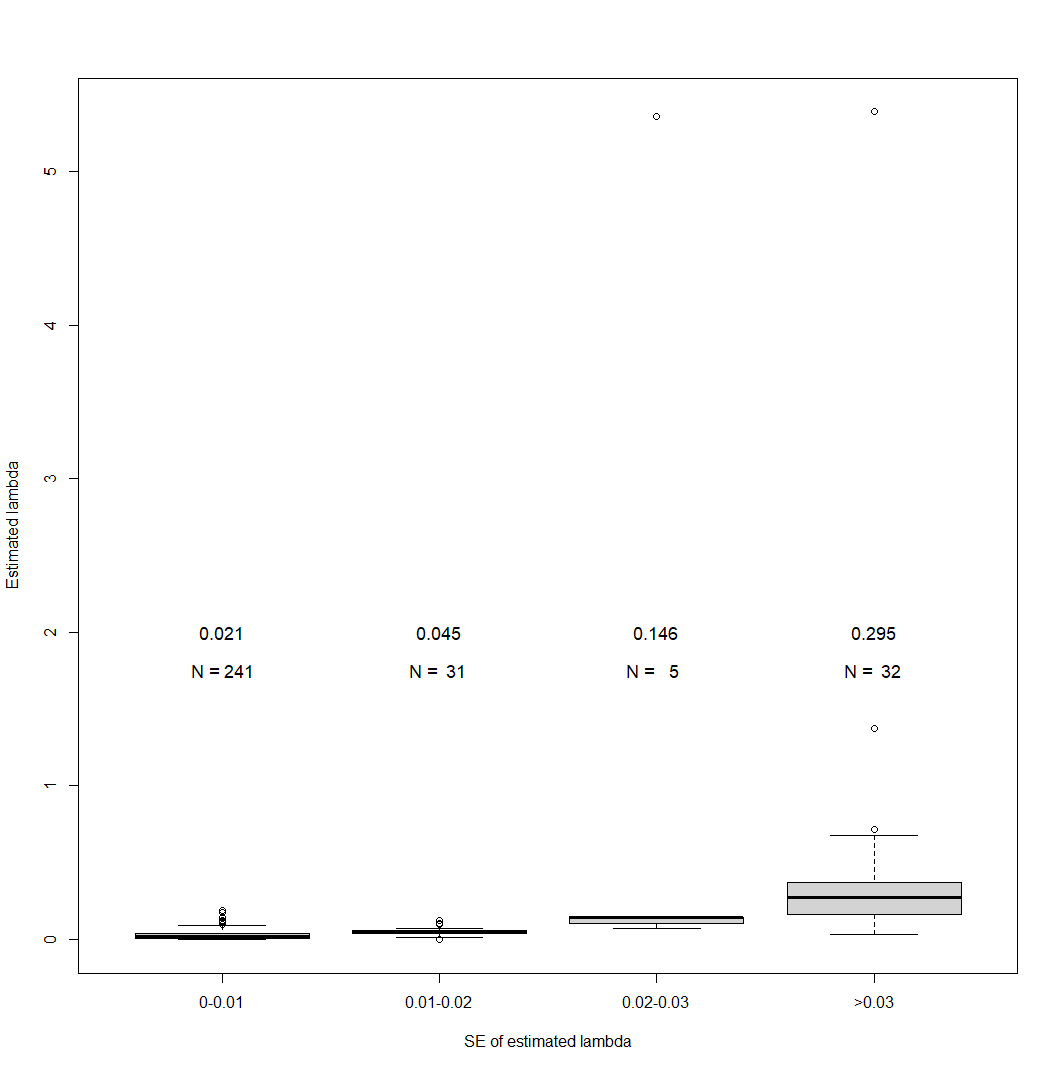


**Fig. S4.** Estimated rate of acclimation (*λ_E_*, h^-1^) for experiments with different levels of uncertainty in estimation (standard error of estimated *λ_E_*). Numbers above each box give the median *λ_E_* and number of experiments for each category.


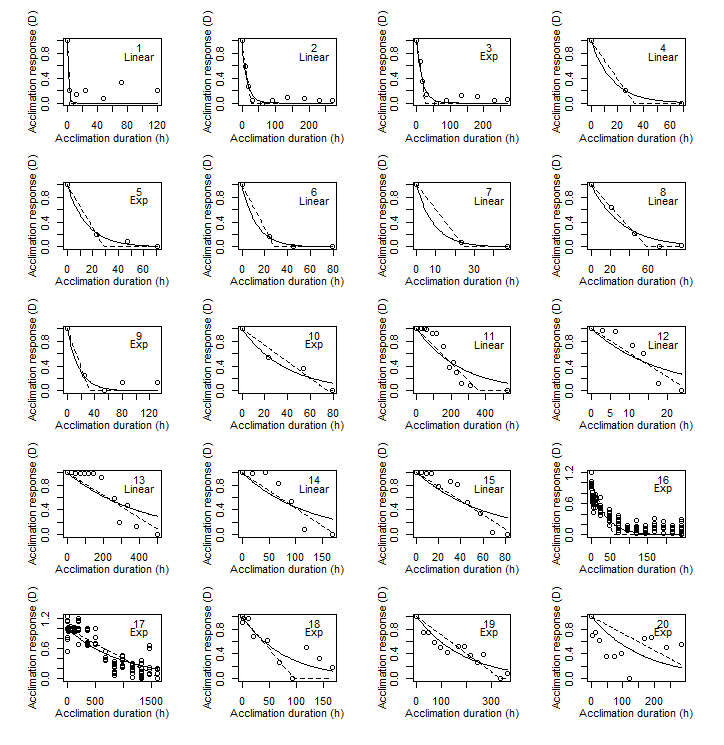


**Fig. S5.** Acclimation response (measured as *D*) as a function of acclimation duration in individual experiments. Data are fitted with segmented regressions (dashed lines, if possible to fit) and exponential decay functions (solid lines). The type of model that gives the best fit (measured as residual errors) is indicated above each panel, as well as experiment number (as given in Table S2).


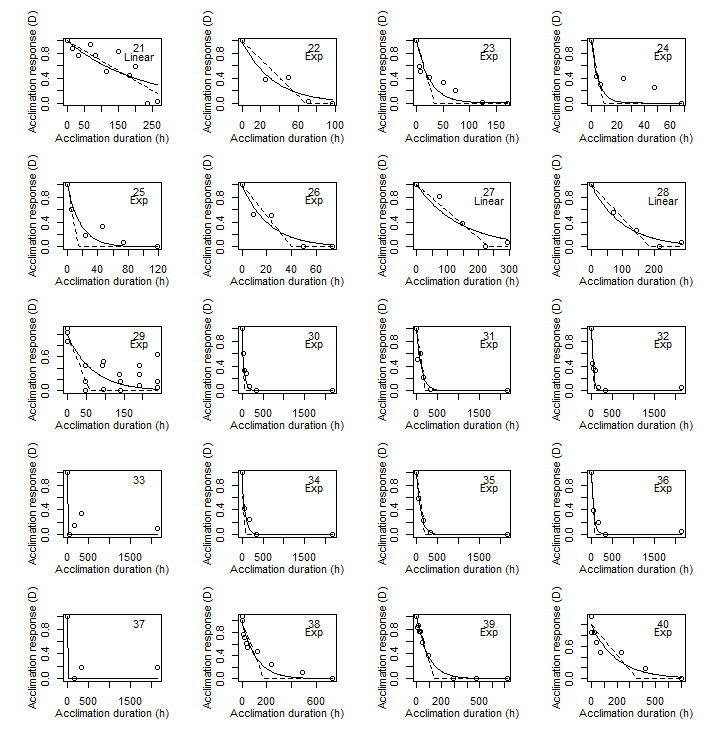


**Fig. S5.** Continued


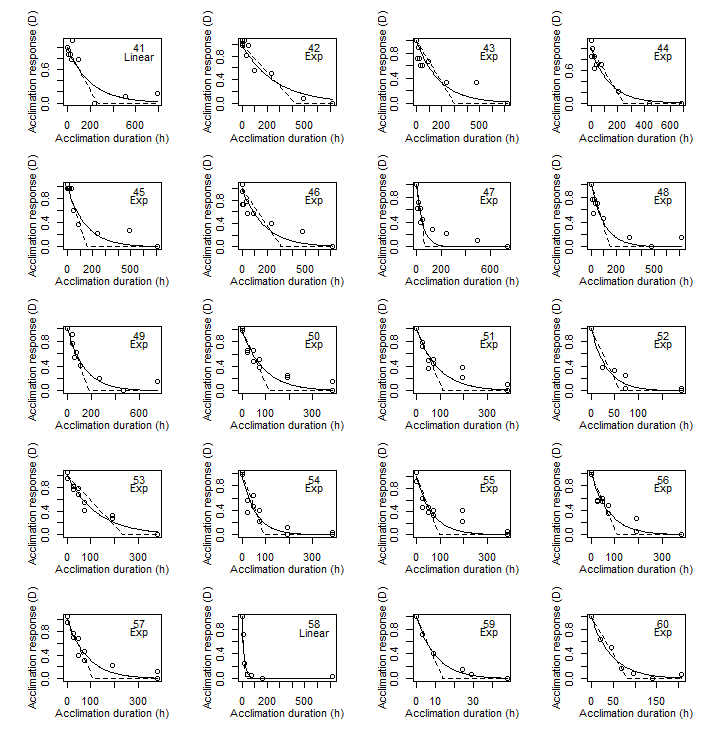


**Fig. S5.** Continued


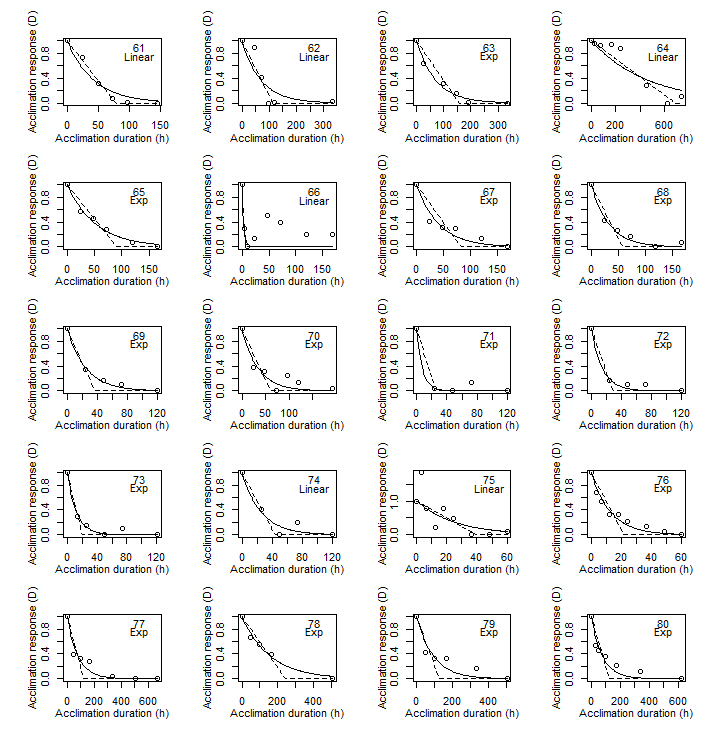


**Fig. S5.** Continued


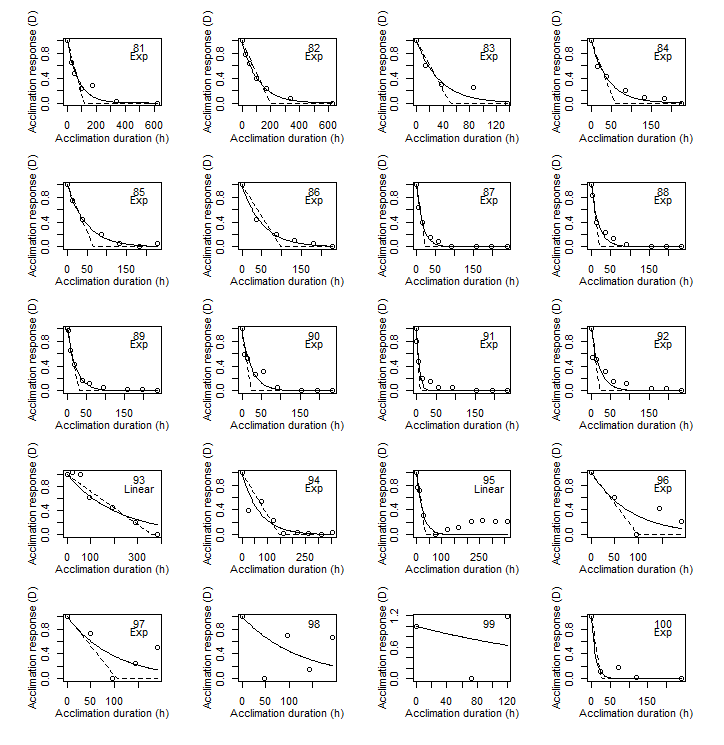


**Fig. S5.** Continued


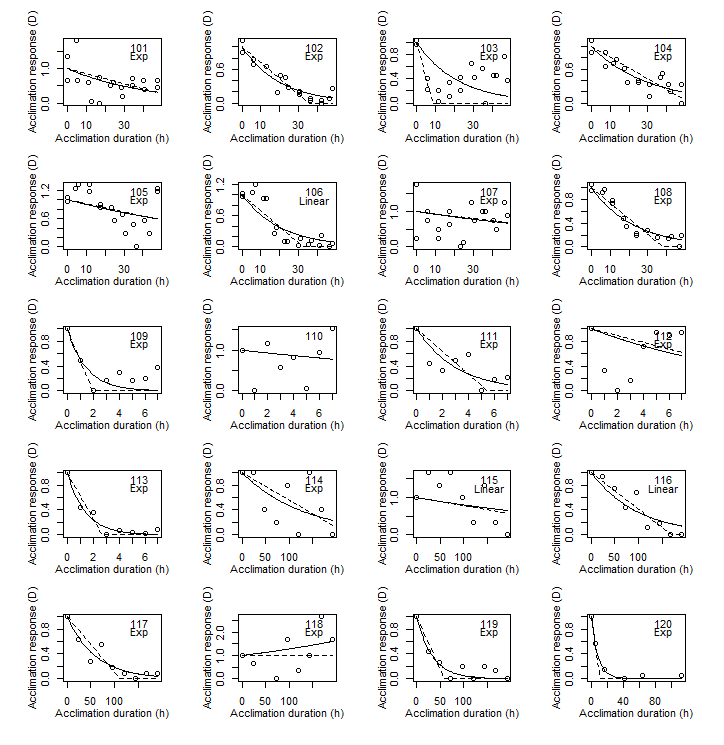


**Fig. S5.** Continued


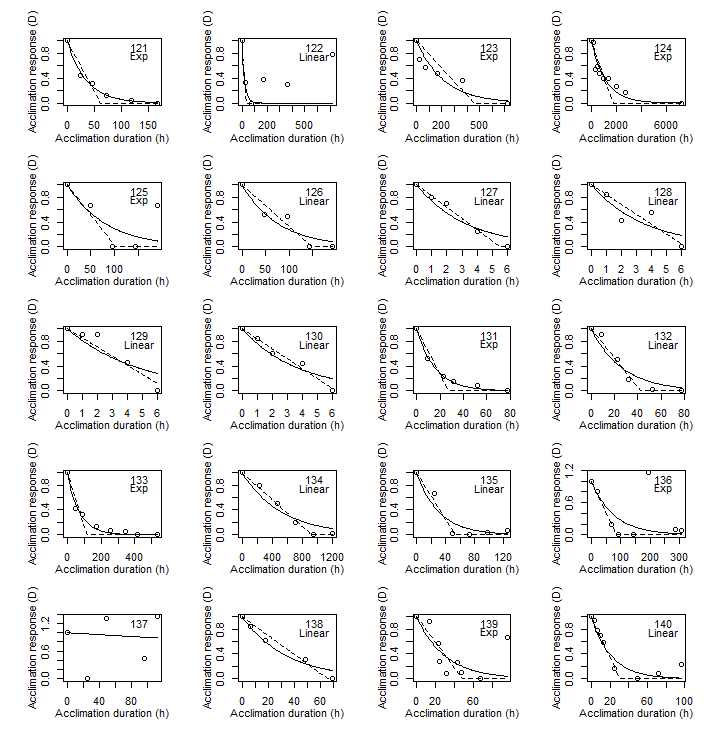


**Fig. S5.** Continued


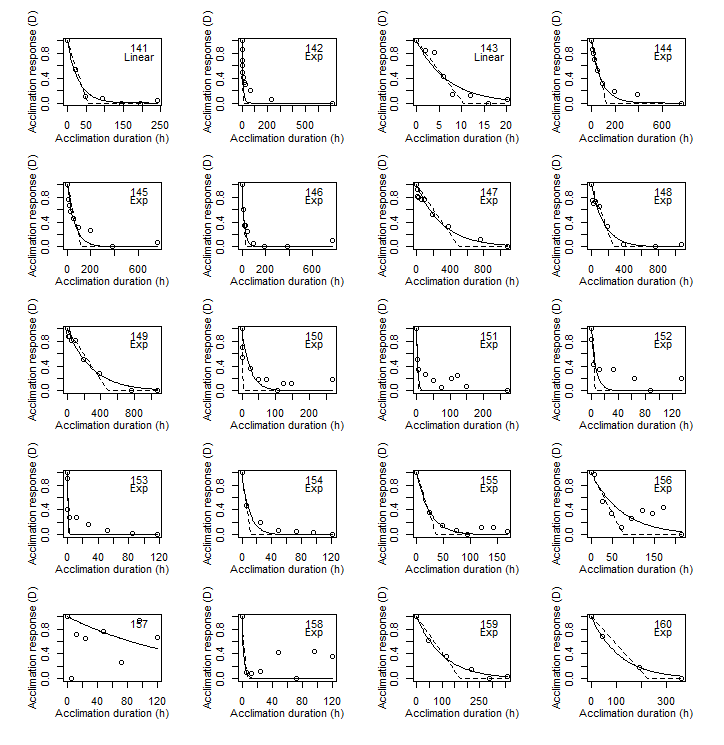


**Fig. S5.** Continued


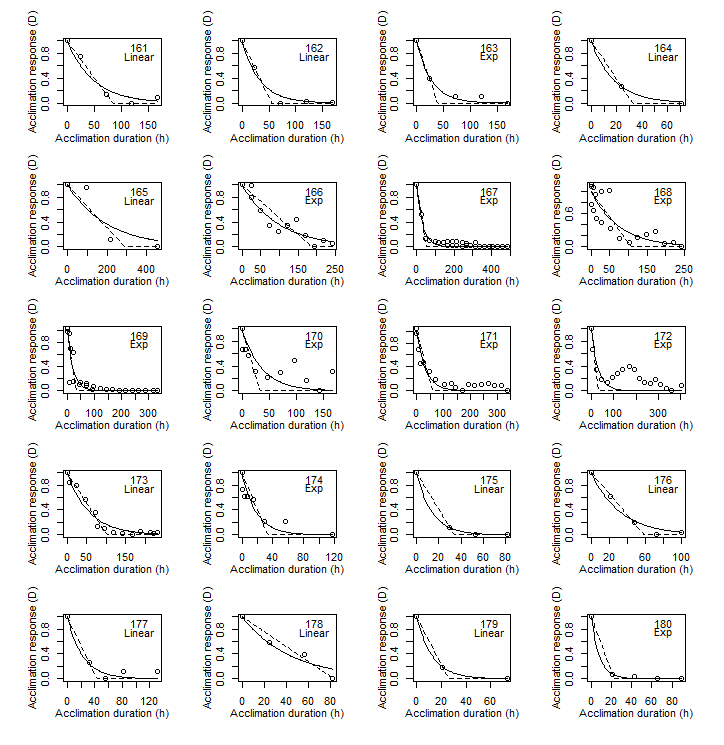


**Fig. S5.** Continued


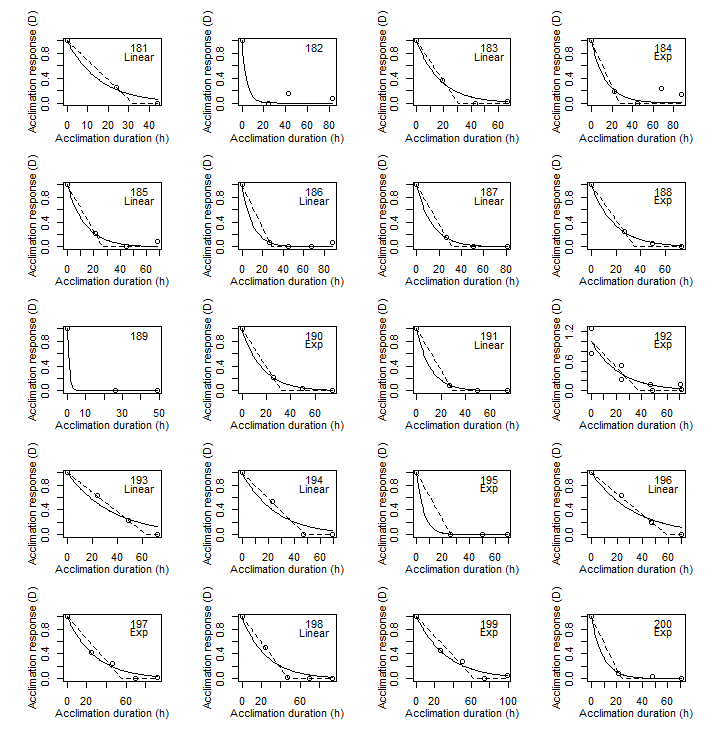


**Fig. S5.** Continued


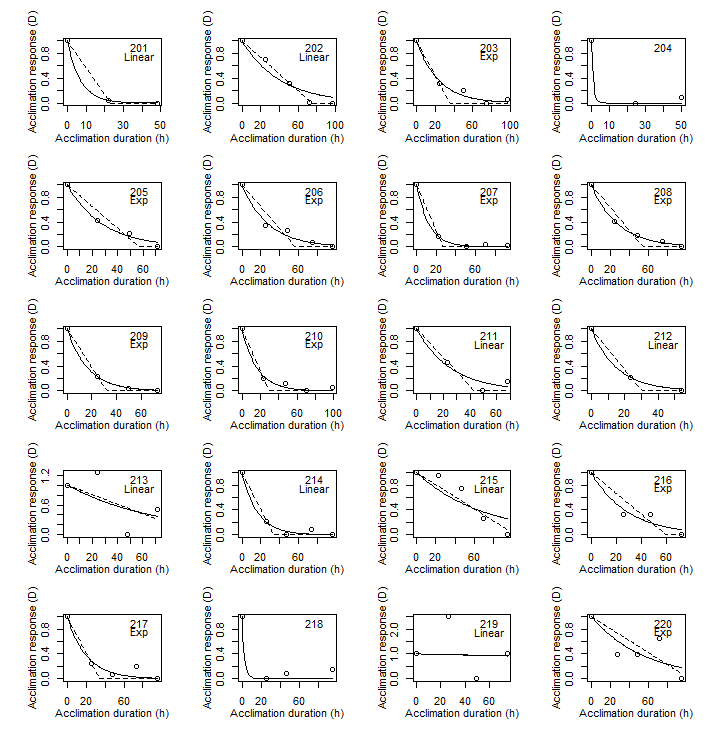


**Fig. S5.** Continued


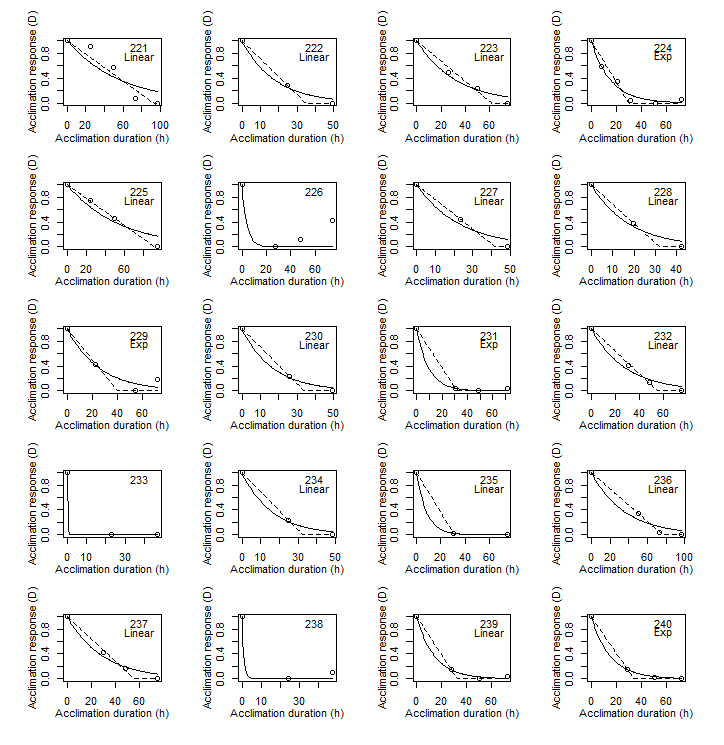


**Fig. S5.** Continued


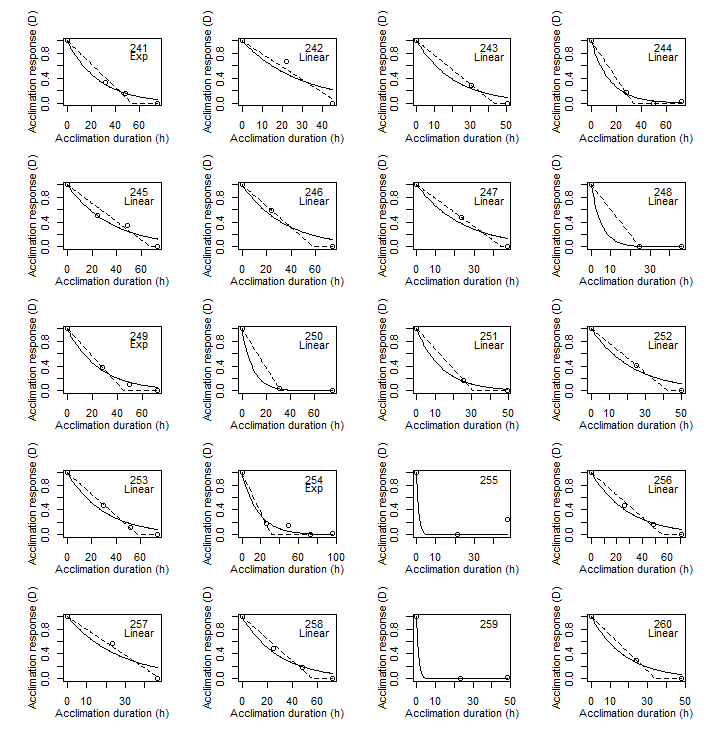


**Fig. S5.** Continued


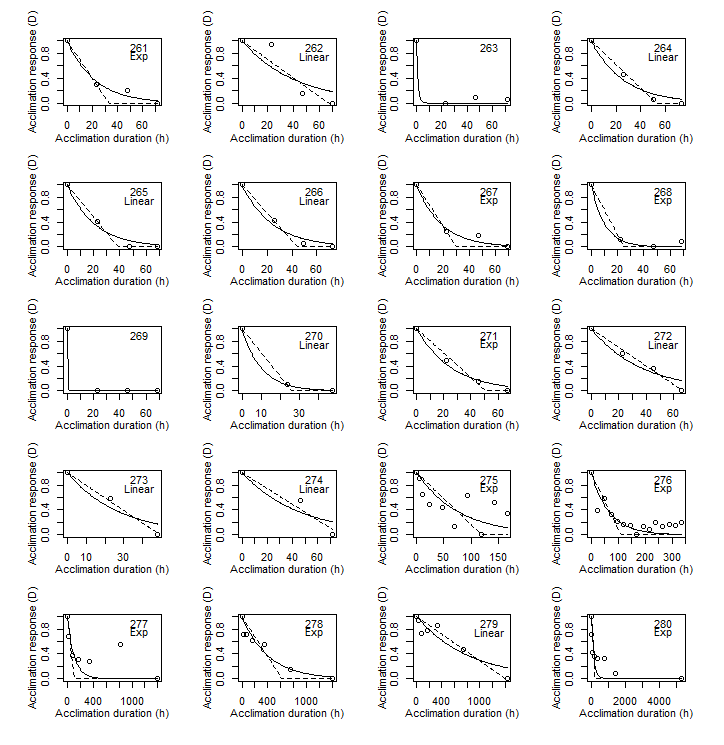


**Fig. S5.** Continued


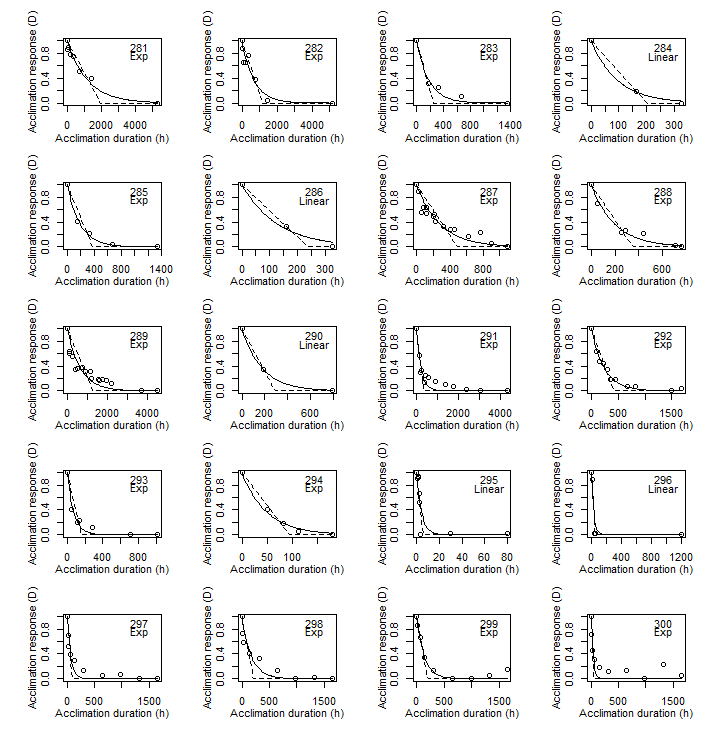


**Fig. S5.** Continued


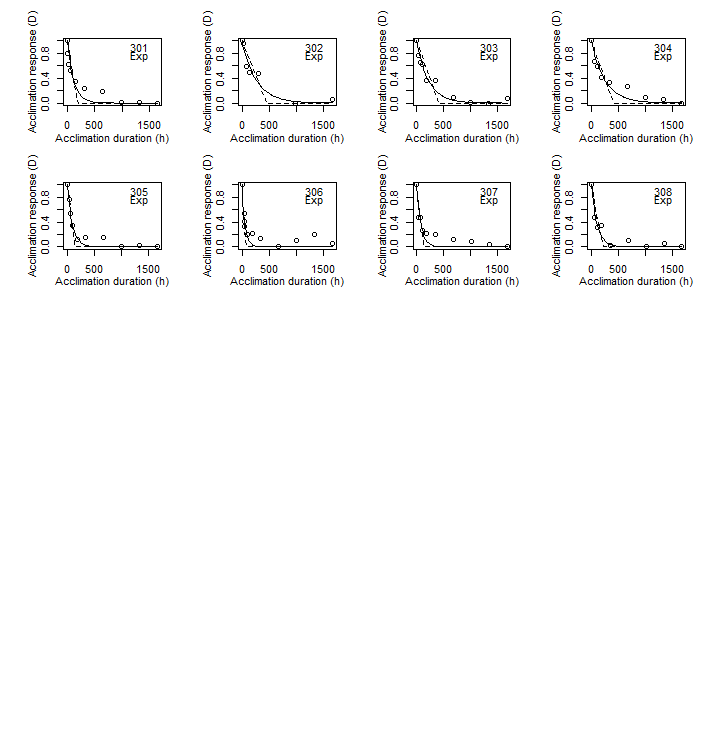


**Fig. S5.** Continued


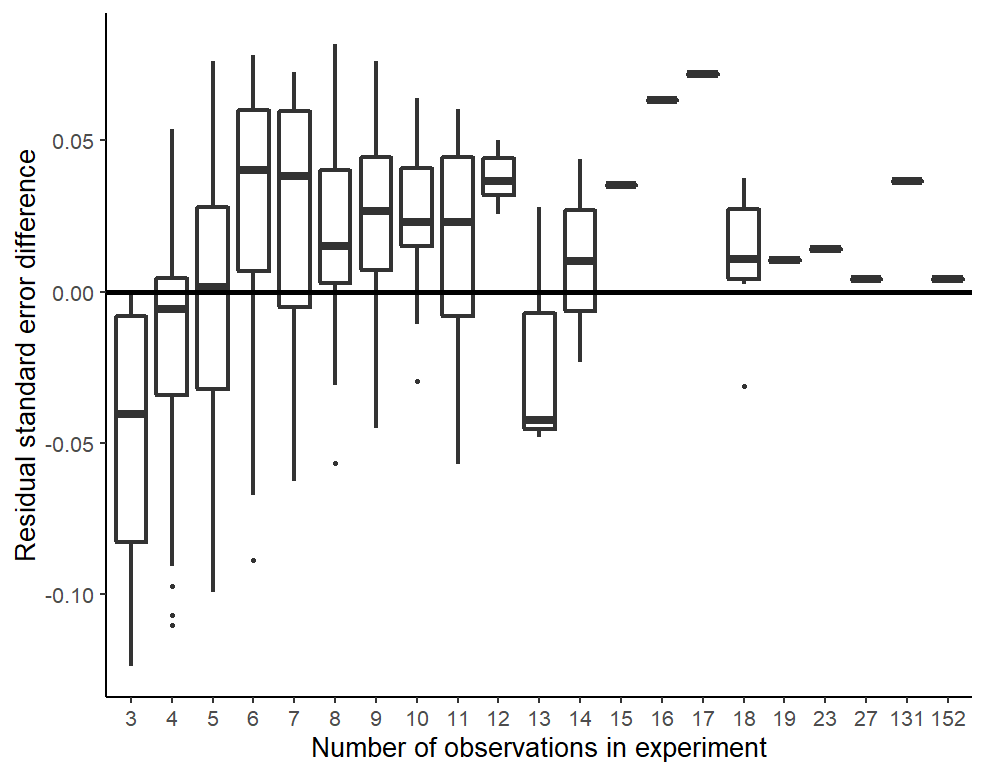


**Fig. S6.** Difference in residual standard error between piecewise regressions and exponential decay functions when fitted to thermal tolerance acclimation data, plotted against number of observations (i.e. measurement time-points) made in experiment. Positive values represent smaller residual errors for fits of exponential decay models than for piecewise regression models.


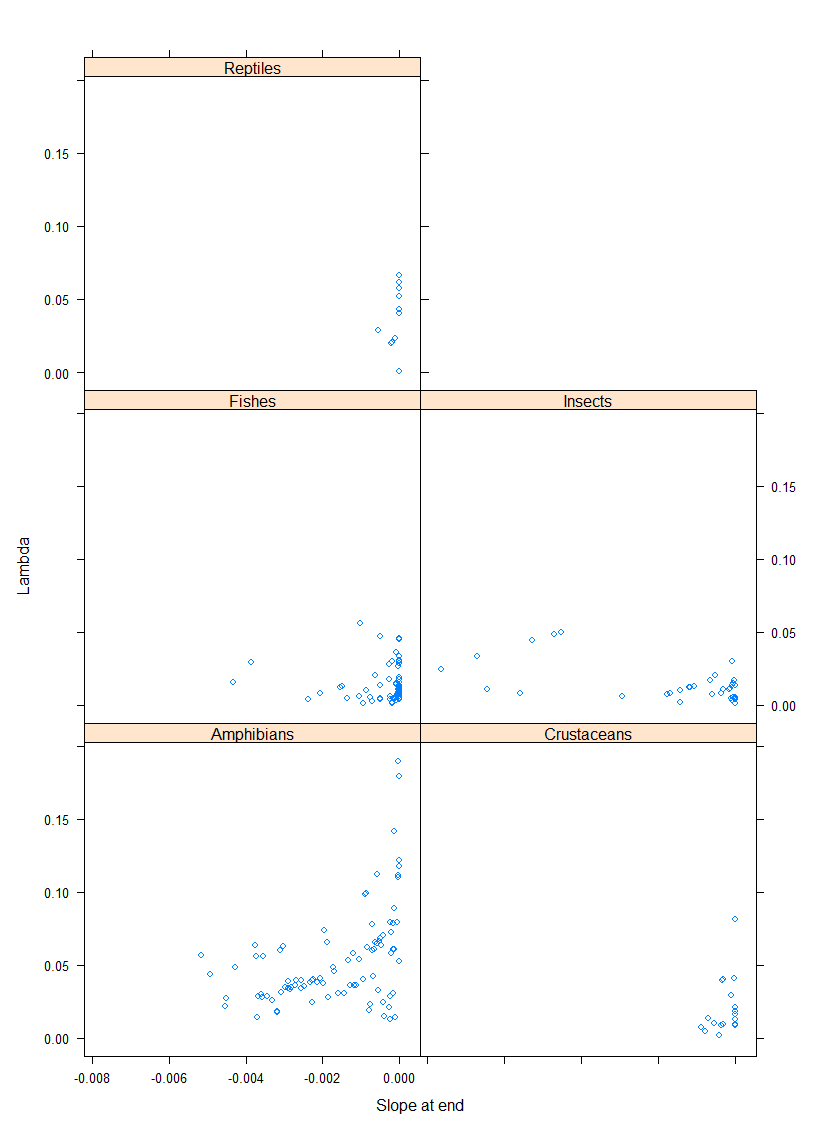


**Fig. S7.** Relationship between slope of the estimated exponential decay function at the final acclimation time point and estimated *λ_E_* (h^-1^).

**Table S1.**1 AICc comparisons of the top candidate models (ΔAIC_C_ < 5) explaining variation in rates of plasticity (*λ_E_*) in temperature tolerance among different classes of ectothermic animals, including data where standard errors for estimates of *λ_E_* < 0.02. ‘Class’ is taxonomic class, 'Slope' is the slope of the estimated exponential decay function at the final measurement, and ‘Measure’ is the measurement type of thermal tolerance (behaviour vs. mortality). Species identity, study and observation are included as random intercepts in all models. For model comparisons based on data where standard errors for estimates of *λ_E_* < 0.01 see Table 1 in the main text.

|  | K | AIC_C_ | ΔAIC_C_ | w_i_ |
| --- | --- | --- | --- | --- |
| Acclimation temperature + Class + Slope + Measure | 11 | -1193.3 | 0.00 | 0.578 |
| Acclimation temperature + Class + Slope | 10 | -1192.6 | 0.63 | 0.421 |

**Table S2.** Estimated rates of acclimation to temperature (*λ_E_*, h^-1^) and associated standard errors. Experiment numbers are the same as those given in Fig. S4. Species names are updated to reflect current taxonomy, and may differ from those given in the original papers. Availability of mass data for inclusion of experiment in the analysis is indicated (Y = yes, N = no), as well as type of measurement (B = behaviour, M = mortality) used as endpoint for thermal tolerance. Note that only experiments where the standard error (SE) of *λ_E_* < 0.01 were included in the analyses due to biased estimates for experiments with larger SE values (see main text). Experiments marked with asterisks were excluded from statistical models due to negative estimate of *λ_E_* (*) missing acclimation temperature data (**), or small species sample size (N = 1-2) at the taxonomic class level (***).

| Exp.nr | Class | Species | *λ_E_* | SE *λ_E_* | Mass  data | Endpoint | Source |
| --- | --- | --- | --- | --- | --- | --- | --- |
| 1 | Reptilia | Anolis carolinensis | 0.5537 | 0.28703 | Y | B | Art & Claussen 1982 |
| 2 | Reptilia | Phrynosoma cornutum | 0.066083 | 0.007316 | Y | B | Ballinger & Schrank 1970 |
| 3 | Reptilia | Phrynosoma cornutum | 0.057742 | 0.006965 | Y | B | Ballinger & Schrank 1970 |
| 4 | Amphibia | Lithobates pipiens | 0.062376 | 0.001861 | Y | B | Brattstrom & Lawrence 1962 |
| 5 | Amphibia | Lithobates pipiens | 0.065293 | 0.004192 | Y | B | Brattstrom & Lawrence 1962 |
| 6 | Amphibia | Lithobates pipiens | 0.078805 | 0.004608 | Y | B | Brattstrom & Lawrence 1962 |
| 7 | Amphibia | Lithobates pipiens | 0.11193 | 0.002095 | Y | B | Brattstrom & Lawrence 1962 |
| 8 | Amphibia | Lithobates clamitans | 0.030811 | 0.004496 | Y | B | Brattstrom & Lawrence 1962 |
| 9 | Amphibia | Lithobates catesbeiana | 0.058419 | 0.014653 | Y | B | Brattstrom & Lawrence 1962 |
| 10 | Amphibia | Lithobates palustris | 0.02609 | 0.004565 | Y | B | Brattstrom & Lawrence 1962 |
| 11 | Osteichtyes | Pimephales promelas | 0.003949 | 0.000681 | Y | M | Brett 1944 |
| 12 | Osteichtyes | Ameiurus nebolosus | 0.054889 | 0.015174 | Y | M | Brett 1944 |
| 13 | Osteichtyes | Carassius auratus | 0.002411 | 0.000542 | Y | M | Brett 1946 |
| 14 | Osteichtyes | Carassius auratus | 0.008147 | 0.002567 | Y | M | Brett 1946 |
| 15 | Osteichtyes | Carassius auratus | 0.015826 | 0.003272 | Y | M | Brett 1946 |
| 16 | Osteichtyes | Cyprinodon dearborni | 0.026827 | 0.00178 | Y | B | Chung 1981 |
| 17 | Osteichtyes | Cyprinodon dearborni | 0.001023 | 5.26E-05 | Y | B | Chung 1981 |
| 18 | Osteichtyes | Astyanax bimaculatus | 0.012583 | 0.002372 | Y | M | Chung 2000 |
| 19 | Osteichtyes | Astyanax bimaculatus | 0.005371 | 0.000559 | Y | M | Chung 2000 |
| 20 | Osteichtyes | Poecilla reticulata | 0.006112 | 0.002063 | Y | M | Chung 2001 |
| 21 | Osteichtyes | Poecilla reticulata | 0.004643 | 0.000975 | Y | M | Chung 2001 |
| 22 | Amphibia | Ambystoma jeffersonianum | 0.030512 | 0.006067 | Y | B | Claussen 1977 |
| 23*** | Turbellaria | Dugesia tigrina | 0.040329 | 0.011048 | Y | B | Claussen 1982 |
| 24*** | Turbellaria | Dugesia dorotocephela | 0.18571 | 0.078648 | Y | B | Claussen 1982 |
| 25*** | Turbellaria | Dugesia tigrina | 0.056584 | 0.016435 | Y | B | Claussen 1982 |
| 26*** | Turbellaria | Dugesia dorotocephala | 0.046537 | 0.010739 | Y | B | Claussen 1982 |
| 27 | Malacostraca | Uca panacea | 0.007113 | 0.00174 | Y | B | Darnell 2015 |
| 28 | Malacostraca | Uca panacea | 0.010016 | 0.001125 | Y | B | Darnell 2015 |
| 29 | Amphibia | Pseudacris triseriata | 0.014996 | 0.003917 | Y | B | Dunlap 1968 |
| 30 | Malacostraca | Porcellio laevis | 0.018686 | 0.001904 | Y | B | Edney 1964 |
| 31 | Malacostraca | Porcellio laevis | 0.008648 | 0.002465 | Y | B | Edney 1964 |
| 32 | Malacostraca | Armadillidum vulgare | 0.020988 | 0.003985 | Y | B | Edney 1964 |
| 33 | Malacostraca | Armadillidum vulgare | 0.668116 | 3.47E+11 | Y | B | Edney 1964 |
| 34 | Malacostraca | Porcellio laevis | 0.013202 | 0.002816 | Y | B | Edney 1964 |
| 35 | Malacostraca | Porcellio laevis | 0.009695 | 0.000525 | Y | B | Edney 1964 |
| 36 | Malacostraca | Armadillidum vulgare | 0.016253 | 0.0033 | Y | B | Edney 1964 |
| 37 | Malacostraca | Armadillidum vulgare | 0.298251 | 4.95E+18 | Y | B | Edney 1964 |
| 38 | Osteichtyes | Cyprinodon variegatus | 0.008815 | 0.001679 | Y | B | Fangue et al. 2014 |
| 39 | Osteichtyes | Cyprinodon variegatus | 0.010797 | 0.000849 | Y | B | Fangue et al. 2014 |
| 40 | Osteichtyes | Cyprinodon variegatus | 0.005078 | 0.00132 | Y | B | Fangue et al. 2014 |
| 41 | Osteichtyes | Cyprinodon variegatus | 0.004884 | 0.001446 | Y | B | Fangue et al. 2014 |
| 42 | Osteichtyes | Cyprinodon variegatus | 0.003806 | 0.000634 | Y | B | Fangue et al. 2014 |
| 43 | Osteichtyes | Cyprinodon variegatus | 0.005595 | 0.001565 | Y | B | Fangue et al. 2014 |
| 44 | Osteichtyes | Cyprinodon variegatus | 0.007243 | 0.001307 | Y | B | Fangue et al. 2014 |
| 45 | Osteichtyes | Cyprinodon variegatus | 0.006898 | 0.001512 | Y | B | Fangue et al. 2014 |
| 46 | Osteichtyes | Cyprinodon variegatus | 0.005772 | 0.00155 | Y | B | Fangue et al. 2014 |
| 47 | Osteichtyes | Cyprinodon variegatus | 0.019449 | 0.004544 | Y | B | Fangue et al. 2014 |
| 48 | Osteichtyes | Cyprinodon variegatus | 0.009535 | 0.002273 | Y | B | Fangue et al. 2014 |
| 49 | Osteichtyes | Cyprinodon variegatus | 0.006987 | 0.000991 | Y | B | Fangue et al. 2014 |
| 50 | Insecta | Drosophila takahashii | 0.011098 | 0.001347 | Y | B | Hori & Kimura 1998 |
| 51 | Insecta | Drosophila takahashii | 0.010988 | 0.001567 | Y | B | Hori & Kimura 1998 |
| 52 | Insecta | Drosophila lutescens | 0.030233 | 0.003271 | Y | B | Hori & Kimura 1998 |
| 53 | Insecta | Drosophila lutescens | 0.007929 | 0.000728 | Y | B | Hori & Kimura 1998 |
| 54 | Insecta | Drosophila rufa | 0.01738 | 0.002577 | Y | B | Hori & Kimura 1998 |
| 55 | Insecta | Drosophila rufa | 0.014912 | 0.002689 | Y | B | Hori & Kimura 1998 |
| 56 | Insecta | Drosophila triauraria | 0.013352 | 0.001608 | N | B | Hori & Kimura 1998 |
| 57 | Insecta | Drosophila triauraria | 0.011859 | 0.001408 | N | B | Hori & Kimura 1998 |
| 58 | Amphibia | Notophthalmus viridescens | 0.052522 | 0.005045 | Y | B | Hutchison 1961 |
| 59 | Amphibia | Notophthalmus viridescens | 0.099219 | 0.005859 | Y | B | Hutchison 1961 |
| 60 | Amphibia | Notophthalmus viridescens | 0.021207 | 0.002225 | Y | B | Hutchison 1961 |
| 61 | Amphibia | Lithobates pipiens | 0.023472 | 0.003794 | Y | B | Hutchison & Michael 1970 |
| 62 | Amphibia | Cryptobranchus alleganiensis | 0.014303 | 0.004524 | Y | B | Hutchison et al. 1973 |
| 63 | Amphibia | Cryptobranchus alleganiensis | 0.014189 | 0.001274 | Y | B | Hutchison et al. 1973 |
| 64 | Malacostraca | Homarus americanus | 0.00216 | 0.00053 | Y | B | Kinne 1964 |
| 65 | Amphibia | Eurycea bislineata | 0.019107 | 0.001367 | Y | B | Layne & Claussen 1982b |
| 66 | Amphibia | Eurycea bislineata | 0.283929 | 0.195511 | Y | B | Layne & Claussen 1982b |
| 67 | Amphibia | Eurycea bislineata | 0.024228 | 0.003867 | Y | B | Layne & Claussen 1982b |
| 68 | Amphibia | Eurycea bislineata | 0.030635 | 0.00261 | Y | B | Layne & Claussen 1982b |
| 69 | Malacostraca | Orconectes rusticus | 0.040527 | 0.002492 | Y | B | Layne et al. 1985 |
| 70 | Malacostraca | Orconectes rusticus | 0.029506 | 0.005723 | Y | B | Layne et al. 1985 |
| 71 | Malacostraca | Orconectes rusticus | 0.129917 | 0.063375 | Y | B | Layne et al. 1985 |
| 72 | Malacostraca | Orconectes rusticus | 0.068217 | 0.01186 | Y | B | Layne et al. 1985 |
| 73 | Malacostraca | Orconectes rusticus | 0.081002 | 0.008523 | Y | B | Layne et al. 1985 |
| 74 | Malacostraca | Orconectes rusticus | 0.039272 | 0.008206 | Y | B | Layne et al. 1985 |
| 75 | Amphibia | Ambystoma tigrinum | 0.039175 | 0.019461 | Y | B | Nietfeldt et al. 1980 |
| 76 | Amphibia | Ambystoma tigrinum | 0.078065 | 0.007314 | Y | B | Nietfeldt et al. 1980 |
| 77 | Osteichtyes | Pagothenia borchgrevinki | 0.012553 | 0.002075 | Y | B | Bilyk & DeVries 2011 |
| 78 | Osteichtyes | Lycodichthys dearborni | 0.006394 | 0.000524 | Y | B | Bilyk & DeVries 2011 |
| 79 | Osteichtyes | Trematomus bernacchii | 0.01072 | 0.002356 | Y | B | Bilyk & DeVries 2011 |
| 80 | Osteichtyes | Lagodon rhomboides | 0.012943 | 0.002183 | Y | B | Reber & Bennett 2007 |
| 81 | Osteichtyes | Lagodon rhomboides | 0.012842 | 0.001719 | Y | B | Reber & Bennett 2007 |
| 82 | Osteichtyes | Lagodon rhomboides | 0.008954 | 0.000223 | Y | B | Reber & Bennett 2007 |
| 83 | Reptilia | Sphenomorphus tympanum | 0.029029 | 0.005444 | Y | B | Spellerberg 1972 |
| 84 | Reptilia | Sphenomorphus tympanum | 0.023419 | 0.002657 | Y | B | Spellerberg 1972 |
| 85 | Reptilia | Sphenomorphus kosciuskai | 0.020595 | 0.00089 | Y | B | Spellerberg 1972 |
| 86 | Reptilia | Sphenomorphus quoyi | 0.019567 | 0.000814 | Y | B | Spellerberg 1972 |
| 87 | Reptilia | Amphibolurus diemensis | 0.061564 | 0.004262 | Y | B | Spellerberg 1972 |
| 88 | Reptilia | Amphibolurus muricatus | 0.051853 | 0.005615 | Y | B | Spellerberg 1972 |
| 89 | Reptilia | Leilopisma guichenoti | 0.043391 | 0.00309 | Y | B | Spellerberg 1972 |
| 90 | Reptilia | Egernia whitei | 0.040623 | 0.008234 | Y | B | Spellerberg 1972 |
| 91 | Reptilia | Egernia saxatilis | 0.14679 | 0.027258 | Y | B | Spellerberg 1972 |
| 92 | Reptilia | Tiliqua rugosa | 0.045859 | 0.011021 | Y | B | Spellerberg 1972 |
| 93 | Malacostraca | Orconectes rusticus | 0.004643 | 0.000877 | Y | B | Spoor 1955 |
| 94 | Osteichtyes | Mugil cephalus | 0.014657 | 0.003056 | Y | M | Sylvester 1974 |
| 95 | Osteichtyes | Mugil cephalus | 0.041036 | 0.012103 | Y | M | Sylvester 1974 |
| 96 | Insecta | Glossina pallidipes | 0.012066 | 0.004087 | Y | B | Terblance et al. 2006 |
| 97 | Insecta | Glossina pallidipes | 0.010158 | 0.004185 | Y | B | Terblance et al. 2006 |
| 98 | Insecta | Glossina pallidipes | 0.008135 | 0.005341 | Y | B | Terblance et al. 2006 |
| 99 | Osteichtyes | Ocyrus chrysurus | 0.003733 | 0.007049 | Y | M | Wallace 1977 |
| 100 | Osteichtyes | Ocyrus chrysurus | 0.086522 | 0.030533 | Y | M | Wallace 1977 |
| 101 | Insecta | Tenebrio molitor | 0.024447 | 0.007528 | Y | B | Allen et al. 2012 |
| 102 | Insecta | Tenebrio molitor | 0.05002 | 0.004578 | Y | B | Allen et al. 2012 |
| 103 | Insecta | Tenebrio molitor | 0.045604 | 0.01285 | Y | B | Allen et al. 2012 |
| 104 | Insecta | Tenebrio molitor | 0.033543 | 0.00349 | Y | B | Allen et al. 2012 |
| 105 | Insecta | Tenebrio molitor | 0.010889 | 0.004381 | Y | B | Allen et al. 2012 |
| 106 | Insecta | Tenebrio molitor | 0.048691 | 0.008739 | Y | B | Allen et al. 2012 |
| 107 | Insecta | Tenebrio molitor | 0.008336 | 0.005339 | Y | B | Allen et al. 2012 |
| 108 | Insecta | Tenebrio molitor | 0.044376 | 0.003843 | Y | B | Allen et al. 2012 |
| 109 | Insecta | Cyrtobagous salviniae | 0.674637 | 0.236567 | Y | B | Allen et al. 2012 |
| 110 | Insecta | Cyrtobagous salviniae | 0.035364 | 0.058585 | Y | B | Allen et al. 2012 |
| 111 | Insecta | Cyrtobagous salviniae | 0.325444 | 0.079999 | Y | B | Allen et al. 2012 |
| 112 | Insecta | Cyrtobagous salviniae | 0.08127 | 0.065577 | Y | B | Allen et al. 2012 |
| 113 | Insecta | Cyrtobagous salviniae | 0.714393 | 0.087483 | Y | B | Allen et al. 2012 |
| 114 | Insecta | Cryptopygus sp. | 0.007564 | 0.002998 | Y | B | Kuyucu & Chown 2021 |
| 115 | Insecta | Cryptopygus sp. | 0.002211 | 0.002273 | Y | B | Kuyucu & Chown 2021 |
| 116 | Insecta | Cryptopygus sp. | 0.010193 | 0.001907 | Y | B | Kuyucu & Chown 2021 |
| 117 | Insecta | Cryptopygus sp. | 0.016983 | 0.002536 | Y | B | Kuyucu & Chown 2021 |
| 118* | Insecta | Mucrosomia caeca | -0.00245 | 0.001564 | Y | B | Kuyucu & Chown 2021 |
| 119 | Insecta | Mucrosomia caeca | 0.03021 | 0.005484 | Y | B | Kuyucu & Chown 2021 |
| 120 | Amphibia | Desmognathus fuscus | 0.117781 | 0.008322 | Y | B | Layne & Claussen 1982a |
| 121 | Amphibia | Desmognathus fuscus | 0.028515 | 0.001617 | Y | B | Layne & Claussen 1982a |
| 122 | Osteichtyes | Oncorhynchus mykiss | 0.046918 | 0.050257 | Y | B | Pandey et al. 2021 |
| 123 | Osteichtyes | Oncorhynchus mykiss | 0.004661 | 0.001114 | Y | B | Pandey et al. 2021 |
| 124 | Reptilia | Carlia longipes | 0.000869 | 0.000104 | Y | B | Pintor et al. 2016 |
| 125 | Insecta | Ceratitis capitata | 0.012227 | 0.00675 | Y | B | Weldon et al. 2011 |
| 126 | Insecta | Ceratitis capitata | 0.012914 | 0.002819 | Y | B | Weldon et al. 2011 |
| 127 | Insecta | Orchesealla cincta | 0.303189 | 0.058593 | Y | B | Alemu et al. 2017 |
| 128 | Insecta | Orchesealla cincta | 0.282458 | 0.079277 | Y | B | Alemu et al. 2017 |
| 129 | Insecta | Orchesealla cincta | 0.211653 | 0.069607 | Y | B | Alemu et al. 2017 |
| 130 | Insecta | Orchesealla cincta | 0.271234 | 0.053297 | Y | B | Alemu et al. 2017 |
| 131 | Amphibia | Chiropterotriton multidentatus | 0.063414 | 0.003515 | Y | B | Brattstrom & Regal 1965 |
| 132 | Amphibia | Chiropterotriton multidentatus | 0.03779 | 0.007146 | Y | B | Brattstrom & Regal 1965 |
| 133 | Osteichtyes | Carassius auratus | 0.01393 | 0.001157 | Y | B | Cossins et al. 1977 |
| 134 | Osteichtyes | Carassius auratus | 0.001925 | 0.000327 | Y | B | Cossins et al. 1977 |
| 135 | Amphibia | Necturus maculatus | 0.032489 | 0.007852 | N | B | Hutchison et al. 1975 |
| 136 | Amphibia | Necturus maculatus | 0.012963 | 0.008514 | N | B | Hutchison et al. 1975 |
| 137 | Osteichtyes | Lycodichthys dearborni | 0.001075 | 0.004295 | Y | M | Podrabsky & Somero 2006 |
| 138 | Osteichtyes | Pagothenia borchgrevinki | 0.029356 | 0.003984 | Y | M | Podrabsky & Somero 2006 |
| 139 | Osteichtyes | Trematomus bernacchii | 0.033865 | 0.01052 | Y | M | Podrabsky & Somero 2006 |
| 140 | Osteichtyes | Mugil curema | 0.047063 | 0.007571 | Y | M | Segnini de Bravo et al. 1993 |
| 141 | Osteichtyes | Mugil curema | 0.033234 | 0.003166 | Y | M | Segnini de Bravo et al. 1993 |
| 142 | Osteichtyes | Gillichthys mirabilis | 0.103839 | 0.02514 | Y | M | Sumner & Doudoroff 1938 |
| 143 | Insecta | Bactrocera tryoni | 0.1456 | 0.024371 | Y | M | Beckett & Evans 1997 |
| 144 | Osteichtyes | Ictalurus punctatus | 0.011624 | 0.001098 | Y | B | Bennett et al. 1998 |
| 145 | Osteichtyes | Ictalurus punctatus | 0.015376 | 0.002692 | Y | B | Bennett et al. 1998 |
| 146 | Osteichtyes | Ictalurus punctatus | 0.045017 | 0.00499 | Y | B | Bennett et al. 1998 |
| 147 | Osteichtyes | Ictalurus punctatus | 0.003277 | 0.000394 | Y | B | Bennett et al. 1998 |
| 148 | Osteichtyes | Ictalurus punctatus | 0.00641 | 0.001119 | Y | B | Bennett et al. 1998 |
| 149 | Osteichtyes | Ictalurus punctatus | 0.003468 | 0.000284 | Y | B | Bennett et al. 1998 |
| 150 | Osteichtyes | Danio rerio | 0.041013 | 0.015632 | Y | B | Aslanidi & Kharakoz 2021 |
| 151 | Osteichtyes | Danio rerio | 0.287349 | 0.089685 | Y | B | Aslanidi & Kharakoz 2021 |
| 152 | Osteichtyes | Danio rerio | 0.140232 | 0.058062 | Y | B | Aslanidi & Kharakoz 2021 |
| 153 | Osteichtyes | Danio rerio | 1.373 | 0.53796 | Y | B | Aslanidi & Kharakoz 2021 |
| 154 | Malacostraca | Paramelita nigroculus | 0.097069 | 0.015918 | N | B | Buchanan et al. 1988 |
| 155 | Malacostraca | Paramelita nigroculus | 0.041173 | 0.005335 | N | B | Buchanan et al. 1988 |
| 156 | Malacostraca | Paramelita nigroculus | 0.013861 | 0.003157 | N | B | Buchanan et al. 1988 |
| 157 | Insecta | Blaptica dubia | 0.0061 | 0.004343 | Y | B | Goode 2013 |
| 158 | Insecta | Blaptica dubia | 0.361887 | 0.390513 | Y | B | Goode 2013 |
| 159 | Malacostraca | Asellus aquaticus | 0.009579 | 0.000579 | Y | B | Lagerspetz & Bowler 1993 |
| 160 | Malacostraca | Asellus aquaticus | 0.008812 | 0.000523 | Y | B | Lagerspetz & Bowler 1993 |
| 161 | Osteichtyes | Cyprinodon macularius | 0.020658 | 0.00409 | Y | M | Lowe & Heath 1969 |
| 162 | Osteichtyes | Cyprinodon macularius | 0.030315 | 0.00484 | Y | M | Lowe & Heath 1969 |
| 163 | Osteichtyes | Cyprinodon macularius | 0.036484 | 0.004779 | Y | M | Lowe & Heath 1969 |
| 164 | Osteichtyes | Cyprinodon macularius | 0.055986 | 0.002074 | Y | M | Lowe & Heath 1969 |
| 165 | Osteichtyes | Oreochromis mossambicus | 0.005034 | 0.002294 | Y | M | Allanson & Noble 1964 |
| 166 | Osteichtyes | Ictalurus punctatus | 0.010231 | 0.001243 | Y | M | Allen & Strawn 1971 |
| 167 | Osteichtyes | Ictalurus punctatus | 0.030758 | 0.002582 | Y | M | Allen & Strawn 1971 |
| 168 | Osteichtyes | Ictalurus punctatus | 0.013347 | 0.002914 | Y | M | Allen & Strawn 1971 |
| 169 | Osteichtyes | Ictalurus punctatus | 0.04592 | 0.008231 | Y | M | Allen & Strawn 1971 |
| 170 | Osteichtyes | Ictalurus punctatus | 0.02772 | 0.009193 | Y | M | Allen & Strawn 1971 |
| 171 | Osteichtyes | Ictalurus punctatus | 0.030028 | 0.00426 | Y | M | Allen & Strawn 1971 |
| 172 | Osteichtyes | Ictalurus punctatus | 0.032418 | 0.010792 | Y | M | Allen & Strawn 1971 |
| 173 | Osteichtyes | Ictalurus punctatus | 0.017862 | 0.00161 | Y | M | Allen & Strawn 1971 |
| 174 | Osteichtyes | Lagodon rhomboides | 0.054465 | 0.013883 | Y | M | Bennett & Judd 1992 |
| 175** | Amphibia | Lithobates pipiens | 0.072841 | 0.003962 | Y | B | Brattstrom 1968 |
| 176** | Amphibia | Lithobates clamitans | 0.031589 | 0.004178 | Y | B | Brattstrom 1968 |
| 177** | Amphibia | Lithobates catesbeiana | 0.044471 | 0.0091 | Y | B | Brattstrom 1968 |
| 178** | Amphibia | Lithobates palustris | 0.022766 | 0.00449 | Y | B | Brattstrom 1968 |
| 179 | Amphibia | Rana pretiosa | 0.079076 | 0.000541 | Y | B | Brattstrom 1968 |
| 180 | Amphibia | Rana cascadae | 0.123799 | 0.01178 | Y | B | Brattstrom 1968 |
| 181 | Amphibia | Rana boylii | 0.063843 | 0.008039 | Y | B | Brattstrom 1968 |
| 182 | Amphibia | Lithobates sylvaticus | 0.227582 | 0.965818 | Y | B | Brattstrom 1968 |
| 183 | Amphibia | Rana pretiosa | 0.058241 | 0.007175 | Y | B | Brattstrom 1968 |
| 184 | Amphibia | Rana cascadae | 0.071404 | 0.027511 | Y | B | Brattstrom 1968 |
| 185 | Amphibia | Rana boylii | 0.074255 | 0.010534 | Y | B | Brattstrom 1968 |
| 186 | Amphibia | Lithobates sylvaticus | 0.098267 | 0.016555 | Y | B | Brattstrom 1968 |
| 187 | Amphibia | Lithobates pipiens | 0.072512 | 0.003856 | Y | B | Brattstrom 1968 |
| 188 | Amphibia | Lithobates pipiens | 0.053918 | 0.001899 | Y | B | Brattstrom 1968 |
| 189 | Amphibia | Lithobates pipiens | 0.887569 | 5159792 | Y | B | Brattstrom 1968 |
| 190 | Amphibia | Lithobates pipiens | 0.060713 | 0.001207 | Y | B | Brattstrom 1968 |
| 191 | Amphibia | Lithobates palmipes | 0.08859 | 0.002829 | Y | B | Brattstrom 1968 |
| 192 | Amphibia | Anaxyrus boreas | 0.044651 | 0.011228 | Y | B | Brattstrom 1968 |
| 193 | Amphibia | Anaxyrus boreas | 0.028311 | 0.005393 | Y | B | Brattstrom 1968 |
| 194 | Amphibia | Anaxyrus boreas | 0.039248 | 0.009875 | Y | B | Brattstrom 1968 |
| 195 | Amphibia | Anaxyrus boreas | 0.179359 | 0.009381 | Y | B | Brattstrom 1968 |
| 196 | Amphibia | Anaxyrus boreas | 0.030097 | 0.006019 | Y | B | Brattstrom 1968 |
| 197 | Amphibia | Anaxyrus boreas | 0.035938 | 0.003335 | Y | B | Brattstrom 1968 |
| 198 | Amphibia | Anaxyrus boreas | 0.040348 | 0.007865 | Y | B | Brattstrom 1968 |
| 199 | Amphibia | Anaxyrus debilis | 0.03086 | 0.003423 | Y | B | Brattstrom 1968 |
| 200 | Amphibia | Incilius alvarius | 0.109843 | 0.007352 | Y | B | Brattstrom 1968 |
| 201 | Amphibia | Anaxyrus fowleri | 0.141646 | 0.00077 | Y | B | Brattstrom 1968 |
| 202 | Amphibia | Rhinella marina | 0.024562 | 0.004989 | Y | B | Brattstrom 1968 |
| 203 | Amphibia | Incilius marmoreus | 0.042583 | 0.004696 | Y | B | Brattstrom 1968 |
| 204 | Amphibia | Incilius mazatlanensis | 0.886437 | 6914438 | Y | B | Brattstrom 1968 |
| 205 | Amphibia | Smilisca fodiens | 0.036042 | 0.003523 | Y | B | Brattstrom 1968 |
| 206 | Amphibia | Hyla walkeri | 0.036245 | 0.003695 | N | B | Brattstrom 1968 |
| 207 | Amphibia | Pseudacris regilla | 0.079486 | 0.006709 | Y | B | Brattstrom 1968 |
| 208 | Amphibia | Pseudacris cadaverina | 0.035835 | 0.001361 | Y | B | Brattstrom 1968 |
| 209 | Amphibia | Pseudacris regilla | 0.060285 | 0.001925 | Y | B | Brattstrom 1968 |
| 210 | Amphibia | Spea hammondii | 0.060954 | 0.007239 | Y | B | Brattstrom 1968 |
| 211 | Amphibia | Spea hammondii | 0.039737 | 0.008164 | Y | B | Brattstrom 1968 |
| 212 | Amphibia | Scaphiopus holbrookii | 0.065279 | 0.004051 | Y | B | Brattstrom 1968 |
| 213 | Amphibia | Anaxyrus boreas | 0.013848 | 0.010811 | Y | B | Brattstrom 1968 |
| 214 | Amphibia | Anaxyrus boreas | 0.06108 | 0.007649 | Y | B | Brattstrom 1968 |
| 215 | Amphibia | Anaxyrus boreas | 0.014392 | 0.004684 | Y | B | Brattstrom 1968 |
| 216 | Amphibia | Anaxyrus boreas | 0.03458 | 0.006805 | Y | B | Brattstrom 1968 |
| 217 | Amphibia | Anaxyrus canorus | 0.049033 | 0.010002 | Y | B | Brattstrom 1968 |
| 218 | Amphibia | Anaxyrus canorus | 0.375099 | 66.03047 | Y | B | Brattstrom 1968 |
| 219** | Amphibia | Anaxyrus exsul | 0.001071 | 0.011909 | Y | B | Brattstrom 1968 |
| 220** | Amphibia | Anaxyrus exsul | 0.018059 | 0.006442 | Y | B | Brattstrom 1968 |
| 221 | Amphibia | Anaxyrus debilis | 0.017488 | 0.005334 | Y | B | Brattstrom 1968 |
| 222 | Amphibia | Incilius alvarius | 0.055827 | 0.007251 | Y | B | Brattstrom 1968 |
| 223 | Amphibia | Lithobates pipiens | 0.031051 | 0.00365 | Y | B | Brattstrom 1968 |
| 224 | Amphibia | Lithobates pipiens | 0.064722 | 0.007905 | Y | B | Brattstrom 1968 |
| 225 | Amphibia | Rana cascadea | 0.018431 | 0.004125 | Y | B | Brattstrom 1968 |
| 226 | Amphibia | Smilisca fodiens | 0.243238 | 6.815415 | Y | B | Brattstrom 1968 |
| 227 | Amphibia | Smilisca fodiens | 0.043635 | 0.010116 | Y | B | Brattstrom 1968 |
| 228 | Amphibia | Tlalocohyla smithii | 0.056432 | 0.009988 | Y | B | Brattstrom 1968 |
| 229 | Amphibia | Pseudacris regilla | 0.039551 | 0.008111 | Y | B | Brattstrom 1968 |
| 230 | Amphibia | Crinia signifera | 0.059919 | 0.006682 | Y | B | Brattstrom 1970 |
| 231 | Amphibia | Crinia signifera | 0.104479 | 0.018325 | Y | B | Brattstrom 1970 |
| 232 | Amphibia | Crinia signifera | 0.035296 | 0.004127 | Y | B | Brattstrom 1970 |
| 233 | Amphibia | Pseudophryne bibronii | 5.389647 | 0.031042 | Y | B | Brattstrom 1970 |
| 234 | Amphibia | Crinia signifera | 0.062546 | 0.00655 | Y | B | Brattstrom 1970 |
| 235 | Amphibia | Crinia signifera | 0.121923 | 0.000103 | Y | B | Brattstrom 1970 |
| 236 | Amphibia | Crinia signifera | 0.027968 | 0.005123 | Y | B | Brattstrom 1970 |
| 237 | Amphibia | Crinia signifera | 0.034243 | 0.003805 | Y | B | Brattstrom 1970 |
| 238 | Amphibia | Geocrinia laevis | 0.944435 | 23834019 | Y | B | Brattstrom 1970 |
| 239 | Amphibia | Pseudophryne bibronii | 0.070505 | 0.005816 | Y | B | Brattstrom 1970 |
| 240 | Amphibia | Pseudophryne bibronii | 0.066359 | 0.001753 | Y | B | Brattstrom 1970 |
| 241 | Amphibia | Pseudophryne bibronii | 0.038523 | 0.002723 | Y | B | Brattstrom 1970 |
| 242 | Amphibia | Philoria frosti | 0.034364 | 0.014632 | Y | B | Brattstrom 1970 |
| 243 | Amphibia | Pseudophryne corroboree | 0.048299 | 0.008956 | Y | B | Brattstrom 1970 |
| 244 | Amphibia | Pseudophryne bibronii | 0.068252 | 0.005186 | Y | B | Brattstrom 1970 |
| 245 | Amphibia | Limnodynastes tasmaniensis | 0.028215 | 0.00455 | Y | B | Brattstrom 1970 |
| 246 | Amphibia | Crinia signifera | 0.028859 | 0.007001 | Y | B | Brattstrom 1970 |
| 247 | Amphibia | Cyclorana brevipes | 0.04192 | 0.011075 | Y | B | Brattstrom 1970 |
| 248 | Amphibia | Cophixalus ornatus | 0.189156 | 0.000492 | Y | B | Brattstrom 1970 |
| 249 | Amphibia | Litoria ewingii | 0.039981 | 0.004223 | Y | B | Brattstrom 1970 |
| 250 | Amphibia | Litoria ewingii | 0.111108 | 0.000172 | Y | B | Brattstrom 1970 |
| 251 | Amphibia | Litoria ewingii | 0.073912 | 0.004833 | Y | B | Brattstrom 1970 |
| 252 | Amphibia | Litoria ewingii | 0.043566 | 0.009474 | Y | B | Brattstrom 1970 |
| 253 | Amphibia | Litoria ewingii | 0.03337 | 0.00551 | Y | B | Brattstrom 1970 |
| 254 | Amphibia | Phyllomedusa bicolor | 0.057946 | 0.00877 | Y | B | Brattstrom 1970 |
| 255 | Amphibia | Phyllomedusa bicolor | 0.924325 | 2881204 | Y | B | Brattstrom 1970 |
| 256 | Amphibia | Phyllomedusa bicolor | 0.03465 | 0.004406 | Y | B | Brattstrom 1970 |
| 257 | Amphibia | Phyllomedusa bicolor | 0.037008 | 0.012708 | Y | B | Brattstrom 1970 |
| 258 | Amphibia | Litoria rubella | 0.034012 | 0.003891 | Y | B | Brattstrom 1970 |
| 259 | Amphibia | Litoria rubella | 0.946458 | 2465722 | Y | B | Brattstrom 1970 |
| 260 | Amphibia | Litoria rubella | 0.056156 | 0.007563 | Y | B | Brattstrom 1970 |
| 261 | Amphibia | Litoria rubella | 0.045889 | 0.006129 | Y | B | Brattstrom 1970 |
| 262 | Amphibia | Litoria rubella | 0.024161 | 0.010606 | Y | B | Brattstrom 1970 |
| 263 | Amphibia | Litoria rothi | 0.908849 | 3422413 | Y | B | Brattstrom 1970 |
| 264 | Amphibia | Litoria peronii | 0.038301 | 0.006158 | Y | B | Brattstrom 1970 |
| 265 | Amphibia | Litoria rothi | 0.048274 | 0.008023 | Y | B | Brattstrom 1970 |
| 266 | Amphibia | Litoria peronii | 0.041256 | 0.005827 | Y | B | Brattstrom 1970 |
| 267 | Amphibia | Ranoidea caerulea | 0.053511 | 0.008254 | Y | B | Brattstrom 1970 |
| 268 | Amphibia | Ranoidea gracilenta | 0.097538 | 0.018209 | Y | B | Brattstrom 1970 |
| 269 | Amphibia | Ranoidea chloris | 5.361701 | 0.025183 | Y | B | Brattstrom 1970 |
| 270 | Amphibia | Ranoidea phyllochroa | 0.098506 | 0.002834 | N | B | Brattstrom 1970 |
| 271 | Amphibia | Ranoidea gracilenta | 0.038862 | 0.004212 | N | B | Brattstrom 1970 |
| 272 | Amphibia | Ranoidea chloris | 0.027377 | 0.005309 | Y | B | Brattstrom 1970 |
| 273 | Amphibia | Ranoidea phyllochroa | 0.036362 | 0.012356 | N | B | Brattstrom 1970 |
| 274 | Amphibia | Ranoidea lesueuri | 0.021904 | 0.008737 | N | B | Brattstrom 1970 |
| 275 | Osteichtyes | Pimelodella chagresi | 0.013087 | 0.003887 | Y | M | Chung 1995 |
| 276 | Osteichtyes | Pimelodella chagresi | 0.01496 | 0.002347 | Y | M | Chung 1995 |
| 277*** | Thecostraca | Elminius modestus | 0.009353 | 0.00455 | N | B | Crisp & Ritz 1967 |
| 278*** | Thecostraca | Elminius modestus | 0.002502 | 0.000529 | N | B | Crisp & Ritz 1967 |
| 279*** | Thecostraca | Elminius modestus | 0.00121 | 0.000281 | N | B | Crisp & Ritz 1967 |
| 280*** | Thecostraca | Elminius modestus | 0.006747 | 0.002203 | N | B | Crisp & Ritz 1967 |
| 281*** | Thecostraca | Elminius modestus | 0.000844 | 0.000106 | N | B | Crisp & Ritz 1967 |
| 282*** | Thecostraca | Elminius modestus | 0.001465 | 0.000325 | N | B | Crisp & Ritz 1967 |
| 283 | Insecta | Sitophilus oryzae | 0.005478 | 0.000804 | Y | B | Evans 1977 |
| 284 | Insecta | Sitophilus oryzae | 0.01061 | 0.000751 | Y | B | Evans 1977 |
| 285 | Insecta | Sitophilus granarius | 0.005206 | 0.000246 | Y | B | Evans 1977 |
| 286 | Insecta | Sitophilus granarius | 0.007825 | 0.001201 | Y | B | Evans 1977 |
| 287 | Insecta | Dacus tryoni | 0.003598 | 0.000339 | Y | B | Meats 1976 |
| 288 | Insecta | Dacus tryoni | 0.005041 | 0.000504 | Y | B | Meats 1976 |
| 289 | Insecta | Dacus tryoni | 0.001533 | 0.000187 | Y | B | Meats 1976 |
| 290 | Insecta | Dacus tryoni | 0.005664 | 0.000122 | Y | B | Meats 1976 |
| 291 | Insecta | Dacus tryoni | 0.004613 | 0.000466 | Y | B | Meats 1976 |
| 292 | Insecta | Dacus tryoni | 0.004113 | 0.000207 | Y | B | Meats 1976 |
| 293 | Insecta | Dacus tryoni | 0.013766 | 0.001615 | Y | B | Meats 1976 |
| 294 | Insecta | Dacus tryoni | 0.020468 | 0.001449 | Y | B | Meats 1976 |
| 295 | Insecta | Dacus tryoni | 0.231905 | 0.07342 | Y | B | Meats 1973 |
| 296 | Insecta | Dacus tryoni | 0.031043 | 0.011869 | Y | B | Meats 1973 |
| 297 | Osteichtyes | Poecilia reticulata | 0.015627 | 0.0028 | Y | B | Tsukuda 1960 |
| 298 | Osteichtyes | Poecilia reticulata | 0.005883 | 0.001856 | Y | B | Tsukuda 1960 |
| 299 | Osteichtyes | Poecilia reticulata | 0.007264 | 0.000822 | Y | B | Tsukuda 1960 |
| 300 | Osteichtyes | Poecilia reticulata | 0.028589 | 0.008906 | Y | B | Tsukuda 1960 |
| 301 | Osteichtyes | Poecilia reticulata | 0.008547 | 0.00204 | Y | B | Tsukuda 1960 |
| 302 | Osteichtyes | Poecilia reticulata | 0.003763 | 0.000723 | Y | B | Tsukuda 1960 |
| 303 | Osteichtyes | Poecilia reticulata | 0.004587 | 0.000532 | Y | B | Tsukuda 1960 |
| 304 | Osteichtyes | Poecilia reticulata | 0.003942 | 0.000577 | Y | B | Tsukuda 1960 |
| 305 | Osteichtyes | Poecilia reticulata | 0.010485 | 0.001147 | Y | B | Tsukuda 1960 |
| 306 | Osteichtyes | Poecilia reticulata | 0.017678 | 0.002906 | Y | B | Tsukuda 1960 |
| 307 | Osteichtyes | Poecilia reticulata | 0.012546 | 0.002245 | Y | B | Tsukuda 1960 |
| 308 | Osteichtyes | Poecilia reticulata | 0.009124 | 0.001178 | Y | B | Tsukuda 1960 |

**Table S3.**1 AICc comparisons of the top candidate models explaining variation in rates of plasticity (*λ_E_*) in temperature tolerance among different classes of ectothermic animals, only including data where the slope of the estimated exponential decay function at the final acclimation time point was larger than -0.002. ‘Class’ is taxonomic class, 'Slope' is the slope of the estimated exponential decay function at the final measurement, and ‘Measure’ is the measurement type of thermal tolerance (behaviour vs. mortality). Species identity, study and observation are included as random intercepts in all models. ΔAIC_C_ = 13.1 for the best model among those not listed.

|  | K | AIC_C_ | ΔAIC_C_ | w_i_ |
| --- | --- | --- | --- | --- |
| Acclimation temperature + Class + Slope + Measure | 11 | -874.4 | 0.00 | 0.500 |
| Acclimation temperature + Class + Slope | 10 | -874.4 | 0.02 | 0.496 |

**Table S4.**1 AICc comparisons of the top candidate models explaining variation in rates of plasticity (*λ_E_*) in temperature tolerance among different classes of ectothermic animals, only including data where the slope of the estimated exponential decay function at the final acclimation time point was larger than -0.001. ‘Class’ is taxonomic class, 'Slope' is the slope of the estimated exponential decay function at the final measurement, and ‘Measure’ is the measurement type of thermal tolerance (behaviour vs. mortality). Species identity, study and observation are included as random intercepts in all models. ΔAIC_C_ = 12.0 for the best model among those not listed.

|  | K | AIC_C_ | ΔAIC_C_ | w_i_ |
| --- | --- | --- | --- | --- |
| Acclimation temperature + Class + Slope | 10 | -737.0 | 0.00 | 0.628 |
| Acclimation temperature + Class + Slope + Measure | 11 | -735.9 | 1.06 | 0.369 |

**Table S5.** Summary of the best fitting linear mixed-effect models from Table S3 (fitted using REML). The amount of variance unaccounted for by fixed effects (*I^2^*, %) explained by the different random effects are given (total *I^2^* = 99.9%). Pseudo-*R^2^* for the model was 0.33.

| Fixed effects | Estimate | 95% CI |
| --- | --- | --- |
| Amphibians | 0.056 | 0.042 – 0.069 |
| Reptiles | 0.028 | 0.006 – 0.051 |
| Insects | 0.012 | -0.006 – 0.029 |
| Fishes | 0.002 | -0.014 – 0.017 |
| Crustaceans | 0.010 | -0.009 – 0.028 |
| Acclimation temperature | 0.0007 | 0.0003 – 0.0010 |
| Slope at end | 23.47 | 16.70 – 30.23 |
| Measurement type (mortality) | 0.012 | -0.005 – 0.029 |
| Random effects | **SD** | ***I^2^*** |
| Study | 0.0002 | 24.2 |
| Species | 0.0003 | 42.6 |
| Observation | 0.0002 | 33.2 |

**Table S6.** Summary of the best fitting linear mixed-effect models from Table S4 (fitted using REML). The amount of variance unaccounted for by fixed effects (*I^2^*, %) explained by the different random effects are given (total *I^2^* = 99.9%). Pseudo-*R^2^* for the model was 0.36.

| Fixed effects | Estimate | 95% CI |
| --- | --- | --- |
| Amphibians | 0.057 | 0.041 – 0.073 |
| Reptiles | 0.027 | 0.002 – 0.052 |
| Insects | 0.006 | -0.015 – 0.027 |
| Fishes | 0.005 | -0.009 – 0.019 |
| Crustaceans | 0.011 | -0.010 – 0.032 |
| Acclimation temperature | 0.0007 | 0.0004 – 0.0011 |
| Slope at end | 27.55 | 15.18 – 39.92 |
| Random effects | **SD** | ***I^2^*** |
| Study | 0.0002 | 29.1 |
| Species | 0.0003 | 45.1 |
| Observation | 0.0002 | 25.8 |

**References**

Alemu, T., Alemneh, T., Pertoldi, C., Ambelu, A., & Bahrndorff, S. (2017). Costs and benefits of heat and cold hardening in a soil arthropod. *Biological Journal of the Linnean Society, 122*(4), 765-773. doi:DOI 10.1093/biolinnean/blx092

Allanson, B. R., & Noble, R. G. (1964). The Tolerance of Tilapia mossambica (Peters) to High Temperature. *Transactions of the American Fisheries Society, 93*(4), 323-332. doi:https://doi.org/10.1577/1548-8659(1964)93[323:TTOTMP]2.0.CO;2

Allen, J. L., Clusella-Trullas, S., & Chown, S. L. (2012). The effects of acclimation and rates of temperature change on critical thermal limits in Tenebrio molitor (Tenebrionidae) and Cyrtobagous salviniae (Curculionidae). *Journal of Insect Physiology, 58*(5), 669-678. doi:10.1016/j.jinsphys.2012.01.016

Allen, K. O., & Strawn, K. (1971). Rate of Acclimation of Juvenile Channel Catfish, Ictalurus-Punctatus, to High Temperatures. *Transactions of the American Fisheries Society, 100*(4), 665-&. doi:Doi 10.1577/1548-8659(1971)100<665:Roaojc>2.0.Co;2

Art, G. R., & Claussen, D. L. (1982). The Rate of Thermal-Acclimation in the Lizard, Anolis-Carolinensis. *Copeia*(1), 189-192. Retrieved from <Go to ISI>://WOS:A1982ND81800029

Aslanidi, K. B., & Kharakoz, D. P. (2021). Limits of temperature adaptation and thermopreferendum. *Cell and Bioscience, 11*(1). doi:10.1186/s13578-021-00574-9

Ballinger, R. E., & Schrank, G. D. (1970). Acclimation Rate and Variability of the Critical Thermal Maximum in the Lizard Phrynosoma cornutum. *Physiological Zoology, 43*(1), 19-22. Retrieved from http://www.jstor.org/stable/30152481

Beckett, S. J., & Evans, D. E. (1997). The effects of thermal acclimation on immature mortality in the Queensland fruit fly Bactrocera tryoni and the light brown apple moth Epiphyas postvittana at a lethal temperature. *Entomologia Experimentalis Et Applicata, 82*(1), 45-51. doi:DOI 10.1046/j.1570-7458.1997.00112.x

Bennett, W. A., & Judd, F. W. (1992). Factors Affecting the Low-Temperature Tolerance of Texas Pinfish. *Transactions of the American Fisheries Society, 121*(5), 659-666. doi:Doi 10.1577/1548-8659(1992)121<0659:Fatlto>2.3.Co;2

Bennett, W. A., McCauley, R. W., & Beitinger, T. L. (1998). Rates of gain and loss of heat tolerance in channel catfish. *Transactions of the American Fisheries Society, 127*(6), 1051-1058. doi:Doi 10.1577/1548-8659(1998)127<1051:Rogalo>2.0.Co;2

Bilyk, K. T., & DeVries, A. L. (2011). Heat tolerance and its plasticity in Antarctic fishes. *Comparative Biochemistry and Physiology a-Molecular & Integrative Physiology, 158*(4), 382-390. doi:10.1016/j.cbpa.2010.12.010

Brattstrom, B. H. (1968). Thermal Acclimation in Anuran Amphibians as a Function of Latitude and Altitude. *Comparative Biochemistry and Physiology, 24*(1), 93-+. doi:Doi 10.1016/0010-406x(68)90961-4

Brattstrom, B. H. (1970). Thermal Acclimation in Australian Amphibians. *Comparative Biochemistry and Physiology, 35*(1), 69-+. doi:Doi 10.1016/0010-406x(70)90915-1

Brattstrom, B. H., & Lawrence, P. (1962). The Rate of Thermal Acclimation in Anuran Amphibians. *Physiological Zoology, 35*(2), 148-156. doi:10.1086/physzool.35.2.30152723

Brattstrom, B. H., & Regal, P. (1965). Rate of Thermal Acclimation in the Mexican Salamander Chiropterotriton. *Copeia, 1965*(4), 514-515. doi:10.2307/1441006

Brett, J. R. (1944). Some lethal temperature relations of Algonquin park fishes. In *University of Toronto studies. Biological series, No. 52*: The University of Toronto Press.

Brett, J. R. (1946). Rate of gain of heat-tolerance in goldfish (Carassius auratus). In *University of Toronto Studies, Biological series, No. 53*: The University of Toronto Press.

Buchanan, J. A., Stewart, B. A., & Davies, B. R. (1988). Thermal-Acclimation and Tolerance to Lethal High-Temperature in the Mountain Stream Amphipod Paramelita-Nigroculus (Barnard). *Comparative Biochemistry and Physiology a-Physiology, 89*(3), 425-431. doi:Doi 10.1016/0300-9629(88)91051-1

Burton, T., Ratikainen, I. I., & Einum, S. (2022). Environmental change and the rate of phenotypic plasticity. Global Change Biology, 28, 5337– 5345. <https://doi.org/10.1111/gcb.16291>

Chung, K. S. (1981). Rate of Acclimation of the Tropical Salt-Marsh Fish Cyprinodon-Dearborni to Temperature-Changes. *Hydrobiologia, 78*(2), 177-181. doi:Doi 10.1007/Bf00007593

Chung, K. S. (1995). Thermal acclimation rate of the tropical long-whiskered catfish Pimelodella chagresi to high temperature. *Carribean Journal of Science, 31*, 154-156.

Chung, K. S. (2000). Heat resistance and thermal acclimation rate in tropical tetra Astyanax bimaculatus of Venezuela. *Environmental Biology of Fishes, 57*(4), 459-463. doi:Doi 10.1023/A:1007696027169

Chung, K. S. (2001). Critical thermal maxima and acclimation rate of the tropical guppy Poecilla reticulata. *Hydrobiologia, 462*, 253-257. doi:Doi 10.1023/A:1013158904036

Claussen, D. L. (1977). Thermal Acclimation in Ambystomatid Salamanders. *Comparative Biochemistry and Physiology a-Physiology, 58*(4), 333-340. Retrieved from <Go to ISI>://WOS:A1977EK06400001

Claussen, D. L., & Walters, L. M. (1982). Thermal-Acclimation in the Fresh-Water Planarians, Dugesia-Tigrina and Dugesia-Dorotocephala. *Hydrobiologia, 94*(3), 231-236. doi:Doi 10.1007/Bf00016404

Cossins, A. R., Friedlander, M. J., & Prosser, C. L. (1977). Correlations between behavioral temperature adaptations of goldfish and the viscosity and fatty acid composition of their synaptic membranes. *Journal of comparative physiology, 120*(2), 109-121. doi:10.1007/BF00619309

Crisp, D. J., & Ritz, D. A. (1967). Temperature acclimation in barnacles. *Journal of Experimental Marine Biology and Ecology, 1*(2), 236-256. doi:https://doi.org/10.1016/0022-0981(67)90017-2

Darnell, M. Z., Nicholson, H. S., & Munguia, P. (2015). Thermal ecology of the fiddler crab Uca panacea: Thermal constraints and organismal responses. *Journal of Thermal Biology, 52*, 157-165. doi:10.1016/j.jtherbio.2015.06.004

Daufresne, M., Lengfellner, K., & Sommer, U. (2009). Global warming benefits the small in aquatic ecosystems. *Proceedings of the National Academy of Sciences, 106*, 12788-12793. doi:doi:10.1073/pnas.0902080106

Dunlap, D. G. (1968). Critical Thermal Maximum as a Function of Temperature of Acclimation in 2 Species of Hylid Frogs. *Physiological Zoology, 41*(4), 432-&. doi:DOI 10.1086/physzool.41.4.30155478

Edney, E. B. (1964). Acclimation to Temperature in Terrestrial Isopods: I. Lethal Temperatures. *Physiological Zoology, 37*(4), 364-377. Retrieved from http://www.jstor.org/stable/30152755

EVANS, D. E. (1977). Some aspects of acclimation to low temperatures in the grain weevils Sitophilus oryzae (L.) and S. granarius (L.). *Australian Journal of Ecology, 2*(3), 309-318. doi:https://doi.org/10.1111/j.1442-9993.1977.tb01147.x

Fangue, N. A., Wunderly, M. A., Dabruzzi, T. F., & Bennett, W. A. (2014). Asymmetric Thermal Acclimation Responses Allow Sheepshead Minnow Cyprinodon variegatus to Cope with Rapidly Changing Temperatures. *Physiological and Biochemical Zoology, 87*(6), 805-816. doi:10.1086/678965

Goode, L. M. (2013). *Effects of thermal accclimation on the critical thermal maxima of the tropical cockroaches: Blaptica dubia, Eublaberus posticus and Blaberus discoidalis (blaberidae).* https://encompass.eku.edu/etd/171.

Hori, Y., & Kimura, M. T. (1998). Relationship between cold stupor and cold tolerance in Drosophila (Diptera : Drosophilidae). *Environmental Entomology, 27*(6), 1297-1302. doi:DOI 10.1093/ee/27.6.1297

Hutchison, V. H. (1961). Critical Thermal Maxima in Salamanders. *Physiological Zoology, 34*(2), 92-125. Retrieved from http://www.jstor.org/stable/30152688

Hutchison, V. H., Engbretson, G., & Turney, D. (1973). Thermal Acclimation and Tolerance in the Hellbender, Cryptobranchus alleganiensis. *Copeia, 1973*(4), 805-807. doi:10.2307/1443083

Hutchison, V. H., & Michael, R. F. (1970). Thermal Tolerances of Rana pipiens Acclimated to Daily Temperature Cycles. *Herpetologica, 26*(1), 1-8. Retrieved from http://www.jstor.org/stable/3891319

Hutchison, V. H., & Rowlan, S. D. (1975). Thermal Acclimation and Tolerance in the Mudpuppy, Necturus maculosus. *Journal of Herpetology, 9*(4), 367-368. doi:10.2307/1562944

Layne, J. R., Manis, M. L., & Claussen, D. L. (1985). Seasonal Variation in the Time Course of Thermal Acclimation in the Crayfish Orconectes rusticus. *Freshwater Invertebrate Biology, 4*(2), 98-104. doi:10.2307/1467181

Kinne, O. (1964). Non-genetic adaptation to temperature and salinity. *Helgoländer wissenschaftliche Meeresuntersuchungen, 9*(1), 433-458. doi:10.1007/BF01610056

Kuyucu, A. C., & Chown, S. L. (2021). Time course of acclimation of critical thermal limits in two springtail species (Collembola). *Journal of Insect Physiology, 130*. doi:ARTN 104209

10.1016/j.jinsphys.2021.104209

Lagerspetz, K. Y. H., & Bowler, K. (1993). Variation in Heat Tolerance in Individual Asellus-Aquaticus during Thermal-Acclimation. *Journal of Thermal Biology, 18*(3), 137-143. doi:Doi 10.1016/0306-4565(93)90027-Q

Layne, J. R., & Claussen, D. L. (1982a). The Time Courses of Ctmax and Ctmin Acclimation in the Salamander Desmognathus-Fuscus. *Journal of Thermal Biology, 7*(3), 139-141. doi:Doi 10.1016/0306-4565(82)90002-X

Layne, J. R., & Claussen, D. L. (1982b). Seasonal-Variation in the Thermal-Acclimation of Critical Thermal Maxima (Ctmax) and Minima (Ctmin) in the Salamander Eurycea-Bislineata. *Journal of Thermal Biology, 7*(1), 29-33. doi:Doi 10.1016/0306-4565(82)90016-X

Lowe, C. H., & Heath, W. G. (1969). Behavioral and Physiological Responses to Temperature in the Desert Pupfish Cyprinodon macularius. *Physiological Zoology, 42*(1), 53-59. Retrieved from http://www.jstor.org/stable/30152465

Meats, A. (1973). Rapid acclimatization to low temperature in the Queensland fruit fly, Dacus tryoni. *Journal of Insect Physiology, 19*(9), 1903-1911. doi:https://doi.org/10.1016/0022-1910(73)90058-9

Meats, A. (1976). Developmental and long-term acclimation to cold by the Queensland fruit-fly (Dacus tryoni) at constant and fluctuating temperatures. *J. Insect Physiol., 22*, 1013-1019.

Nietfeldt, J. W., Jones, S. M., Droge, D. L., & Ballinger, R. E. (1980). Rate of Thermal Acclimation in Larval Ambystoma tigrinum. *Journal of Herpetology, 14*(3), 209-211. doi:10.2307/1563541

O'Dea, R.E., Lagisz, M., Jennions, M.D., Koricheva, J., Noble, D. W. A., Parker, T. H. *et al.* (2021) Preferred reporting items for systematic reviews and meta-analyses in ecology and evolutionary biology: a PRISMA extension. *Biological Reviews*, 96, 1695-1722. doi:https://doi.org/10.1111/brv.12721

Pandey, A., Rajesh, M., Baral, P., Sarma, D., Tripathi, P. H., Akhtar, M. S., . . . Kamalam, B. S. (2021). Concurrent changes in thermal tolerance thresholds and cellular heat stress response reveals novel molecular signatures and markers of high temperature acclimation in rainbow trout. *Journal of Thermal Biology, 102*. doi:ARTN 103124

10.1016/j.jtherbio.2021.103124

Pintor, A. F. V., Schwarzkopf, L., & Krockenberger, A. K. (2016). Extensive Acclimation in Ectotherms Conceals Interspecific Variation in Thermal Tolerance Limits. *Plos One, 11*(3). doi:ARTN e0150408

10.1371/journal.pone.0150408

Podrabsky, J. E., & Somero, G. N. (2006). Inducible heat tolerance in Antarctic notothenioid fishes. *Polar Biology, 30*(1), 39-43. doi:10.1007/s00300-006-0157-y

Reber, C. M., & Bennett, W. A. (2007). The influence of thermal parameters on the acclimation responses of pinfish Lagodon rhomboides exposed to static and decreasing low temperatures. *Journal of Fish Biology, 71*(3), 833-841. doi:10.1111/j.1095-8649.2007.01552.x

Segnini de Bravo, M. I., Chung, K. S., & Ciurcina, P. (1993). Tasa de aclimatacion al cambio de temperatura de Mugil curema (Pisces: Mugilidae) de Venezuela. *Rev. Biol. Trop., 41*, 59-62.

Spellerberg, I. F. (1972). Temperature Tolerances of Southeast Australian Reptiles Examined in Relation to Reptile Thermoregulatory Behavior and Distribution. *Oecologia, 9*(1), 23-+. doi:Doi 10.1007/Bf00345241

Spoor, W. A. (1955). Loss and Gain of Heat-Tolerance by the Crayfish. *Biological Bulletin, 108*(1), 77-87. doi:10.2307/1538399

Sumner, F. B., & Doudoroff, P. (1938). Some Experiments upon Temperature Acclimatization and Respiratory Metabolism in Fishes. *Biological Bulletin, 74*(3), 403-429. doi:10.2307/1537814

Sylvester, J. R. (1974). Thermal Response of Juvenile Hawaiian Mullet Mugil-Cephalus (L) to Acclimation Time and Fluctuating Low-Temperatures. *Journal of Fish Biology, 6*(6), 791-796. doi:DOI 10.1111/j.1095-8649.1974.tb05121.x

Terblanche, J., Klok, C. J., Krafsur, E. S., & Chown, S. L. (2006). Phenotypic plasticity and geographic variation in thermal tolerance and water loss of the tsetse Glossina pallidipes (Diptera : Glossinidae): Implications for distribution modelling (vol 74, pg 786, 2006). *American Journal of Tropical Medicine and Hygiene, 75*(1), 186-186. Retrieved from <Go to ISI>://WOS:000238902600034

Tsukuda, H. (1960). Temperature adaptation in fishes IV. Change in the heat and cold tolerances of the Guppy in the process of temperature acclimatization. *Journal of the Institute of Polytchnics*, 43-54.

Wallace, R. K. (1977). Thermal Acclimation, Upper Temperature Tolerance, and Preferred Temperature of Juvenile Yellowtail Snappers, Ocyurus-Chrysurus (Bloch) (Pisces - Lutjanidae). *Bulletin of Marine Science, 27*(2), 292-298. Retrieved from <Go to ISI>://WOS:A1977DE56100007

Weldon, C. W., Terblanche, J. S., & Chown, S. L. (2011). Time-course for attainment and reversal of acclimation to constant temperature in two Ceratitis species. *Journal of Thermal Biology, 36*(8), 479-485. doi:10.1016/j.jtherbio.2011.08.005
